# A beneficial bacterium mitigates drought stress by upregulating the flavonoid biosynthetic pathway in *Arabidopsis*

**DOI:** 10.1080/15592324.2026.2639558

**Published:** 2026-03-10

**Authors:** Roy N. Kimotho, Emma W. Gachomo

**Affiliations:** a Department of Microbiology and Plant Pathology, University of California, Riverside, Riverside, CA, USA

**Keywords:** Plant growth promoting rhizobacteria, PGPR, priming, anthocyanin, chemotaxis, *Pantoea* species

## Abstract

Drought stress hinders plant growth and causes major yield losses globally. This study investigated the mechanisms behind *Pantoea* species-induced drought tolerance in *Arabidopsis*. RNA-seq analyses revealed significant upregulation of the flavonoid biosynthesis pathway in *Pantoea*-inoculated plants under drought stress. The *Pantoea* strain, which is a plant growth-promoting rhizobacteria (PGPR), led to a significant increase in chlorophyll content and enhanced drought tolerance in wild-type *Arabidopsis*, but not in flavonoid pathway mutants (*fls-1*, *tt4-2*, and *omt1*), demonstrating the role of flavonoids in the interaction. Inoculation significantly upregulated flavonoid biosynthesis genes, including MYB11, *CHI*, *CHS*, *F3H*, *F3'H*, and *FLS1,* and significantly increased the flavonoid and anthocyanin contents in wild type plants compared to the mutants under drought stress, thus confirming that flavonoids are involved in *Pantoea*-induced drought stress. Wild type had higher colonization than the mutants, implicating flavonoids in root colonization. *Pantoea* species swarmed towards flavonoid soaked-agar plugs suggesting that flavonoids may be chemoattractants. To our knowledge, this is the first time that the flavonoid biosynthesis pathway has been implicated in *Pantoea*-induced drought tolerance. Overall, our findings revealed that *Pantoea-*mediated drought stress tolerance is largely regulated by the flavonoid biosynthesis pathway, providing insights into the role of flavonoids in plant–microbe interactions and drought stress tolerance.

## Introduction

Abiotic stresses such as drought, heat, salinity and erratic water availability are the leading causes of reduced crop yields globally and are estimated to account for 50%–60% of potential agricultural production losses.^1^ Drought alone was responsible for approximately 34% of crop and livestock production losses in low- and middle-income countries between 2008 and 2018, and ~$30 billion in crop production losses globally in the past decade.^2^^,1^ With extreme global temperatures and shifts in weather patterns, abiotic stresses have become frequent key constraints on crop production, thereby exacerbating food insecurity. Drought, in particular, is a potent abiotic stressor that affects ecosystem functions such as plant growth and production.^3^ Drought also has significant impacts on the soil microbiota, often leading to decreased microbial biomass.^4^ The intensifying frequency of drought in many regions of the world necessitates innovative and sustainable strategies to ensure stable and reliable food production.^5^^,^^6^

As sessile organisms, plants have a limited ability to move in response to abiotic stressors.^7^ Therefore, they adapt to drought conditions through different strategies, including avoidance or escape (e.g., stomatal closure, leaf rolling, and wax deposition) and recovery (e.g., slower growth rates and partial decline in functionality).^8^ They also employ tolerance mechanisms such as osmolyte adjustment, phytohormone signaling, and antioxidant defenses.^8^ Antioxidant defenses involve both enzymes (such as superoxide dismutase, ascorbate peroxidase, and glutathione reductase) and nonenzymatic compounds, including polyphenols, terpenoids, and carotenoids.^8^ Examples of polyphenols are flavonoids. They are a diverse group of secondary metabolic compounds that are widely distributed in the plant kingdom and they play a crucial role in plant growth, development, and regulation of biotic and abiotic stress.^9^ Flavonoids are classified into six major categories – flavones, flavonols, anthocyanins, flavanols, flavanones, and isoflavones.^9^^,^^10^ They accumulate in plants under different abiotic stresses such as nutrient deficiency (nitrogen and phosphate), heat, salinity, and drought.^9^^,^^11-13^ Flavonoids are important as signaling molecules, antioxidants, protectors of cells from oxidative injury, and facilitators of faster recovery from stress.^9^ In addition, they have been implicated in plant-microbe interactions, playing vital roles in rhizosphere microbiome community composition and dynamics.^9^^,^^14^^,^^15^ Flavonoids mediate the communication between plants and plant growth-promoting rhizobacteria (PGPR), underscoring their significance in these interactions.^16^ For example, inoculation with *Pseudomonas fluorencens* G20-18 has been reported to increase antioxidant enzymes, and secondary metabolites like phenolics, flavonoids, and anthocyanins under drought.^17^ Anthocyanins produced downstream of flavonoids are also known to reduce oxidative stress resulting from drought stress.^18^

Plant–microbe associations have played a key role in plant growth, immune responses, and performance throughout plant evolution.^19^^,^^20^ PGPR, which are often used as biostimulants and biocontrol agents,^21^^,^^22^ increase plant growth and development. They achieve this either directly by producing phytohormones (indole-3-acetic acid, ethylene, and cytokinins) and providing essential nutrients (phosphorus, nitrogen, and iron) or indirectly by inducing the production of hormones, osmolytes, and other protective metabolites.^23^^-^^25^ PGPR have been reported to enhance plant growth and survival under drought conditions.^26^^,^^27^ In addition, PGPR induce systemic resistance (ISR) against plant pathogens, thereby enhancing plant defense systems.^28^^,^^29^ Among the different responses elicited by PGPR, the upregulation of stress-associated metabolic pathways has garnered significant interest. Under drought conditions, PGPR employ different mechanisms to improve plant growth. For example, the regulation of abscisic acid, which is vital for stomatal closure during water stress,^30^ enhances root growth and root surface augmentation, allowing plants to access deeper water reserves,^31^ the production of osmolytes that maintain cell turgor during drought, and the stimulation of antioxidant production in response to reactive oxygen species (ROS)^32^^,^^33^). Further, PGPR assist in solubilizing nutrients, thus increasing nutrient availability, as it is usually reduced under drought stress.^31^ Consequently, they enhance plant nutrient acquisition and survival under drought stress. Therefore, understanding the mechanisms used by PGPR to increase drought stress tolerance is essential for their selection and deployment in sustainable agriculture.

Our preliminary experiments showed that a *Pantoea* strain induced drought stress tolerance in plants, raising our interest in the underlying mechanism. Subsequent RNA-seq analysis led us to hypothesize that *Pantoea* confers drought tolerance to plants by upregulating the flavonoid biosynthesis pathway. Although many studies have examined PGPR-induced production of secondary metabolites in plants, none have specifically addressed the interplay between flavonoid biosynthesis, PGPR colonization, and drought stress tolerance. To address this gap, our work provides an analysis of the mechanisms used by *Pantoea* species to confer drought stress tolerance in *Arabidopsis*, with a focus on flavonoids. By integrating transcriptomics, molecular, physiological and biochemical approaches, we highlight the interactions among PGPR, plant flavonoid/anthocyanin biosynthesis, and drought stress. We showed that *Pantoea* species colonization induced the expression of key flavonoid biosynthesis genes and enhanced flavonoid synthesis under drought conditions. By comparing Col-0 (wild-type) with several flavonoid biosynthetic pathway mutants (*tt4-2*, *fls1-3*, and *omt1*), our study revealed that flavonoids are required for the colonization of *Arabidopsis* by *Pantoea* species.

## Materials and methods

### Plant materials and growth conditions

#### Arabidopsis thaliana

Wild-type plants (Col-0) and the flavonoid biosynthesis mutants *fls1-3*, *tt4-2*, and *omt1* in the Col-0 background were used in this study. The mutant seeds were kindly provided by Gloria Muday.^34^ All the seeds were surface sterilized using 3% sodium hypochlorite for 10 min, followed by three thorough rinses with sterile water. Seeds were germinated on filter papers with evenly spaced holes on solid ½ strength Murashige and Skoog (MS) media containing 1.5% sucrose and 1% agar. Seeds were vernalized at 4 °C for 72 h before being transferred to a growth chamber with 70% relative humidity, 150 μmol m⁻² s⁻¹ light intensity, a 12-h light/dark photoperiod, and 22 °C day and 21 °C night temperatures. Additional plants were grown in soil (Sunshine Mix #1 by Sun Gro® Horticulture − USA) under similar conditions in a growth room.

### Bacterial strain and inoculum preparation

Sanger sequencing analysis of the 16S rRNA gene of the bacteria used in this study showed that this isolate shares 100% sequence identity with *Pantoea* species. From a glycerol stock stored at −80 °C, and the bacterium was streaked on Luria–Bertani (LB) agar plates and incubated for 24 h. A single colony was inoculated into 10 mL of LB broth and grown for 24 h at 28 °C and 200 rpm. This overnight culture was used to make 100 mL of culture that was grown under the same conditions until it reached the exponential phase, which was estimated by having an optical density of 0.8–1.2 at a wavelength of 600 nm (OD600). The bacterial broth was centrifuged at 4500 rpm for 15 min, and the pellet was resuspended in 10 mM MgSO_4_ solution (an isotonic solution that prevents bacterial cell plasmolysis or damage). The OD600 was adjusted to 1.0 before inoculation of the growth media in the hydroponic tanks or soil in the pots.

### Plant growth in hydroponic tanks

*The Arabidopsis* seeds were surface sterilized, vernalized, germinated on filter papers on MS agar, and placed in a growth chamber as described above. Seven-day-old seedlings on filter papers were aseptically transferred to hydroponic tanks containing 150 mL of full-strength MS liquid media supplemented with 1.5% sucrose. Each tank was equipped with a mesh barrier that separated the shoots from the liquid media, ensuring that only the roots were in contact with the media. The tanks and MS media were autoclaved prior to transfer of the seedlings. The tanks were wrapped with Parafilm and placed in a growth chamber under similar conditions as those described above, and the plants were allowed to grow for another 7 d before inoculating the growth media with either *Pantoea* species in MgSO_4_ or sterile MgSO_4_, which served as the treatment and control, respectively. After 7 d, the seedlings were transferred into fresh MS media supplemented with 20% (w/v) polyethylene glycol 6000 (PEG) to induce drought stress. Fresh tissues were collected at 6, 12, 24, and 48 h and at 7 d post-drought stress. The plant tissue was used for gene expression analysis, flavonoid and anthocyanin quantification, and RNA sequencing. Plants from five tanks (20 plants) were collected to form one biological replicate. For recovery from drought stress, *Arabidopsis* seedlings were transferred from MS media with 20% PEG supplemented with 1.5% sucrose to tanks containing fresh full-strength MS media supplemented with 1.5% sucrose (recovery media). Subsequently, samples were collected at 6, 12, 24, and 48 h and 7 d after being placed in the recovery media for gene expression analysis and quantification of flavonoids and anthocyanins. The experiment had four treatments: control [plants grown in MS media inoculated with MgSO_4_ and later transferred to fresh MS media], control + PGPR [plants grown in MS media inoculated with *Pantoea* species suspended in MgSO_4_ and later transferred to fresh MS media], control + PEG [plants grown in MS media inoculated with MgSO_4_ and later transfer to MS containing 20% (w/v) PEG], and PGPR + PEG [plants grown in MS media inoculated with *Pantoea* species suspended in MgSO_4_ and later transferred to MS containing 20% PEG] (Table 1). Five biological replicates were included for all the treatments, and the experiments were repeated three times.

**Table 1.** Treatment composition.

Treatment	Inoculation medium	Transfer medium
Control	MS media + MgSO₄	Fresh MS media
Control + PGPR	MS media + *Pantoea* species in MgSO₄	Fresh MS media
Control + PEG	MS media + MgSO₄	MS media containing 20% PEG
PGPR + PEG	MS media + *Pantoea* species in MgSO₄	MS media containing 20% PEG

### Drought stress of pot grown plants

Ten-day-old *Arabidopsis* seedlings growing in autoclaved soil (Sunshine Mix #1 by Sun Gro® Horticulture, USA) in a growth room were inoculated with *Pantoea* species, and the bacteria were allowed to establish in the soil for 10 d. The growth conditions were: 21 °C–23 °C, 16 h light/8 h dark (long-day), 120–150 µmol m⁻² s⁻¹ (cool white fluorescent), and relative humidity of 40%–60%. The plants were then subjected to drought by withholding water 10 d post inoculation. The soil volumetric water content was monitored daily using a plant hygrometer moisture sensor. Drought treatment reduced the volumetric water content from 100% at day 0 to 5% at day 10, and this level was maintained by a combination of pot weighing and sensor monitoring daily. For well-watered conditions, plants were maintained under a regular watering schedule, with 820 mL of distilled water added to individual trays every 96 h (referred to as drought stress and well-watered conditions, respectively, in the manuscript). There were 24 plants per treatment in each of the independent experiments, which were arranged in a completely randomized design. After 10 d of drought stress, the plants were rewatered, and the percentage of recovery of Col-0 and the mutant plants was determined over the next 10 d. All the plants that did not recover after 10 d of rewatering were considered dead. Five days into drought stress, photos of the plants were taken from above and used to measure the diameter of the rosette using ImageJ (NIH).^35^ In addition, some plants were used to measure shoot biomass, and leaf tissues were collected to assay chlorophyll content.

### Chlorophyll measurements

One hundred milligrams of fresh leaves were collected and incubated overnight in 2.5 mL of 80% acetone at 4 °C. Thereafter, the mixture was centrifuged at 12,000 rpm at room temperature, and the supernatant was collected in a new, clean tube. The absorbance spectra of the supernatants were measured at wavelengths of 647 and 664 nm. The chlorophyll content was calculated based on the following formula: 20.31 × A_645_ × 8.05 × A_663_/FW (µg g^−1^).^36^

### RNA-seq

Total RNA was extracted from whole *Arabidopsis* wild-type (Col-0) plants grown in hydroponic tanks and subjected to drought stress as described in section above, using the GeneJet Kit (GeneJET Plant RNA Purification Kit; Thermo Scientific, Madison, WI, USA) according to the manufacturer's instructions.

In preparation for RNA-seq, the RNA was processed (74204 QIAGEN and AM1907 Invitrogen), and the NEBNext Ultra Directional RNA Library Prep Kit was used to prepare the libraries for Illumina following the manufacturer's protocol (E7490S, E7335S, and E7420S; New England Biolabs). Pooled libraries were sequenced on the Illumina NextSeq 500 platform (Illumina, San Diego, CA, USA) at the University of California–Riverside Genomics Core Facility.

To quantify the transcript data from all the samples, the raw RNA-seq data were processed using the program Salmon^37^ and then analyzed using the R program ThreeDRNA.^38^^-^^40^ Briefly, the integrated tximport program (v. 1.18.0) and the default setting lengthScaledTPM method were used to upload the quantified transcript data into ThreeDRNA. The default count per million reads cutoff of 1 and sample number cutoff of 1 were used to filter low-quality expression data. Then PCA graphs were generated using the RUVr method of removing batch effects from the data. The weighted trimmed means of the M values in ThreeDRNA were used to normalize the data. Statistical analysis was done using the limma-voom pipeline.^41^^,^^42^ Samples that did not have *p* values ≤0.05 and log_2_ FC ≥1 between the treatments were filtered out.

To further characterize the transcriptional reprogramming of *Arabidopsis* in response to inoculation with *Pantoea* species under drought, we performed Gene Ontology (GO) and Kyoto Encyclopedia of Genes and Genomes (KEGG) enrichment analyses on the differentially expressed genes (DEGs). GO annotations (biological process, cellular component and molecular function) were identified for *A.* *thaliana* genes based on the Gene Ontology database.^43^ While KEGG pathway annotations were retrieved from the KEGG database.^44^ Further analysis was carried out using the clusterProfiler R package GO terms and KEGG pathways with an adjusted *p* value (FDR) <0.05 and a minimum gene count (e.g., ≥5 DEGs per term/pathway) were considered significantly enriched.

### In vitro ROS Detection using DCFDA Staining

Ten-day-old *Arabidopsis* Col-0 plants were grown on ½ MS media and inoculated with *Pantoea* sp. (OD = 1) suspended in 10 mM MgSO, and mock controls received sterile 10 mM MgSO₄. The seedlings were maintained on ½ MS for 7 d and then transferred to PEG-containing medium to induce drought stress. Subsequently, roots were harvested and stained with 2ʹ,7ʹ-dichlorodihydrofluorescein diacetate (H2DCFDA) to visualize the accumulation of reactive oxygen species (ROS). The root samples were carefully excised 48 h after transfer to PEG and washed three times with 1× phosphate-buffered saline (PBS) to remove residual media and unattached bacteria. The roots were then completely submerged in a 10 µM H2DCFDA working solution, which was freshly prepared in the dark. The samples were incubated in the dark at room temperature for 30 min to allow the dye to fully penetrate the tissues and be oxidized into highly fluorescent 2ʹ,7ʹ-dichlorofluorescein (DCF) by intracellular ROS. Following incubation, the roots were washed three additional times with 1× PBS to remove any unbound dye. The stained roots were immediately mounted on glass slides and imaged using a fluorescence microscope using a standard GFP/FITC filter set (excitation ~488 nm; emission ~520–525 nm). Image processing and channel merging were performed using Fiji to assess the spatial distribution and relative intensity of the ROS signal.

### Quantitative real time PCR (qRT-PCR)

*Arabidopsis* seeds of the wild type (Col-0) were germinated on filter papers and transferred to hydroponic tanks as described above. Whole plant tissues from wild-type *Arabidopsis* (Col-0) were collected at 6, 12, 24, and 48 h after the start of either the PEG or recovery treatments and immediately stored at −80 °C until RNA was extracted as described above. Two micrograms of total RNA were used for reverse transcription to synthesize cDNA. Genomic DNA was removed using DNase I and RNase. Subsequently, qRT-PCR was performed using 96 well plates with a Bio-Rad CFX96 Touch Real-Time PCR System (Bio-Rad) as previously described by Nam et al.^36^ Relative gene expression levels were calculated using the 2^−ΔΔCt^ method, with the *Ubiquitin* 10 gene (AT4g05320) used as the housekeeping gene. All primers used in this study are listed in Supplementary Table 1. For gene expression quantification, relative transcript levels were compared between the control and PGPR-inoculated samples at each time point using a two-way ANOVA. Tukey's post hoc test was used for pairwise comparisons to assess statistical significance between treatment groups at each time point. The data are presented as mean ± SEM. *p* values less than 0.05 were considered statistically significant. The experiment had a total of 30 plants per treatment and three biological replicates in each treatment.

### Quantification of flavonoids and anthocyanins

#### Flavonoids

Two hundred milligrams of frozen tissues were extracted in 2 mL of 80% methanol for 2.5 h with shaking (200 rpm) at 4 °C as described by Sharma et al.^45^^,^^46^ The mixture was subsequently centrifuged at 13,500 rpm for 10 min at 4 °C. Thereafter, 0.5 mL of the supernatant was mixed with 2 mL of 100% methanol, 100 µl of 1 M potassium acetate, 100 µl of a 10% aluminum chloride solution (water), and 2.8 mL of distilled water. The samples were incubated for 30 min at room temperature, and the absorbance was recorded at 415 nm.

#### Anthocyanins

For anthocyanin quantification, the tissues stored at −80 °C were freeze dried using a freeze dryer (Labconco 700201000 FreeZone) and ground into a fine powder using a GenoGrinder (HG-600 Geno/Grinder). One hundred milligrams of the lyophilized tissues were transferred to a freshly prepared solution of propanol:HCl:H_2_O at a ratio of 18:1:18 according to the methods of Sharma et al.^45^ The samples were subsequently heated in a water bath at 95 °C for 3 min and incubated in the dark at room temperature for 2 h. Thereafter, the mixture was centrifuged at 13,500 rpm for 15 min at 4 °C, and the supernatant was collected. The absorbance of the samples was measured at 535 nm and 650 nm. The anthocyanin concentrations were calculated using the following formula:^45^

$$(A_{535}-{2.2A}_{650})/\mathrm{g FW}.$$

#### Root colonization by *Pantoea* species

Ten-day-old Col-0 and flavonoid mutant plants grown in sterile soil were inoculated with *Pantoea* sp. As previously described, the control plants were inoculated with sterile MgSO_4._ After 10 d, the plants were subjected to drought for 5 d and thereafter the plants were rewatered. Fresh roots were harvested from soil-grown plants at different time points before, during and after drought stress. One hundred milligrams of freshly harvested roots were placed in a tube with 1000 µL of sterile 10 mM MgSO_4_, and the samples were homogenized using a glass rod and mixed thoroughly using a vortex machine at full speed. Serial dilutions were performed using sterile 10 mM MgSO_4_, and 10 µl of the resulting solution was spread on LB agar plates to determine the concentration of bacteria. The plates were incubated overnight at 30 °C, the resulting bacterial colonies were counted, and the results are expressed as CFU/g of fresh weight. For the in vitro system, 10-d-old *Arabidopsis* Col-0 and flavonoid pathway mutants were grown and inoculated with *Pantoea* sp. (OD = 0.001), mock controls received sterile 10 mM MgSO₄. Seedlings were maintained on ½ MS for 7 d, transferred to PEG-containing media to induce drought stress for 5 d, and subsequently transferred to ½ MS for recovery. Roots were harvested aseptically at the indicated time points before, during, and after stress. Roots were weighed (100 mg fresh weight per sample), placed in sterile tubes containing 1000 µL sterile 10 mM MgSO₄ and subjected to brief sonication to detach loosely associated bacteria. Each biological replicate contained fresh roots from 50 seedlings. The roots were then homogenized using sterile glass rods to release the remaining bacteria, and the homogenate was mixed thoroughly. Serial dilutions were prepared in sterile 10 mM MgSO₄, and 10 µL of appropriate dilutions was spread on LB agar. The plates were incubated overnight at 30 °C, colonies were counted, and the bacterial abundance was expressed as CFU g⁻¹ fresh root weight.

#### *Pantoea* species chemotaxis towards flavonoids

After observing that root colonization was significantly higher in Col-0 than in the flavonoid mutants, the chemotaxis of *Pantoea* sp. toward flavonoids was assayed on 1.5% water agar plates following a modified method by Ye et al. ^47^ Briefly, quercetin and kaempferol were dissolved in methanol to a final concentration of 100 µM. Subsequently, sterile 5-mm agar plugs were soaked in 500 µL of filter-sterilized quercetin or kaempferol for 1 h at room temperature, after which the plugs were placed gently on the agar surface. Ten microliters of a dense exponentially growing *Pantoea* sp. The suspension (OD₆₀₀ 0.5) was spot inoculated 2 cm away from the plugs containing quercetin and kaempferol. Plates with agar plugs soaked in filter-sterilized methanol for 1 h served as controls. The plates were incubated at 30 °C for 0–24  h, after which the growth of the *Pantoea* sp. bacteria toward the plug was analyzed using a DM 2500 microscope (Leica, Buffalo Grove, IL). The experiment had three replicates and was repeated three times.

### Statistical analysis

For all the experiments with quantitative data, the mean values are presented with corresponding standard deviations. One-way analysis of variance (ANOVA) was used to test the significance between three groups or more using the Tukey's post hoc test. Unpaired two-tailed Student's *t* test was used to test for significance between two groups. In all tests, *p* < 0.05 was considered to be statistically significant. All the statistical analyses and corresponding graphs were created using GraphPad Prism Software v9 (GraphPad Software, Boston, MA).

## Results

### *Pantoea* species changed the transcriptome of *Arabidopsis* plants under drought stress

**Figure 1.**
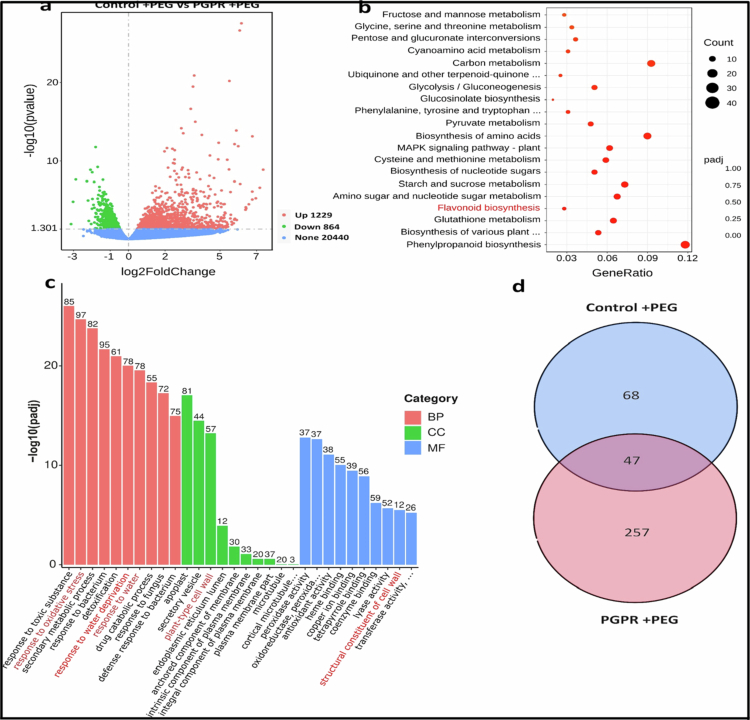
RNA sequencing data: (a) volcano plots showing DEGs in the control + PEG vs PGPR + PEG treatments with more genes being upregulated than downregulated. The total number of upregulated and downregulated genes were 1229 and 854, respectively. Each dot represents DEGS with expression changed more than 1.5-fold (*p* < 0.05). (b) KEGG terms in the control + PEG vs PGPR + PEG treatments. The dot size indicates the number of DEGs. (c) GO enrichment of upregulated DEGs in PGPR + PEG compared with the control + PEG treatment group. The enriched GO terms are shown and grouped by GO domain (BP, biological process; CC, cellular component; and MF, molecular function). The bar height indicates enrichment significance as –log_10_, and the numbers above the bars denote the number of upregulated genes annotated to each GO term. (d) Venn diagram showing overlapping DEGS in the control + PEG vs PGPR + PEG treatment groups. The experiment had four treatments, with 30 plants per treatment and three biological replicates. *n* = 120. DEGs = differentially expressed genes, PGPR = plant growth-promoting rhizobacteria, and PEG = polyethylene glycol 6000. The PGPR used in this study is a *Pantoea* strain.

Our preliminary experiments revealed that the *Pantoea* strain used in the study enhanced drought tolerance and induced the expression of drought-responsive genes in *Arabidopsis*. However, the underlying mechanisms have not been elucidated; therefore, we performed a transcriptome analysis of *Arabidopsis* wild-type (Col-0) plants treated with polyethylene glycol 6000 (PEG 6000) to simulate drought stress for 5 d after inoculation with or without *Pantoea* sp. The experiment consisted of two treatments, namely, control + PEG and PGPR + PEG. RNA sequencing data analysis identified 2093 differentially expressed genes (DEGs) in drought-stressed plants that were either inoculated or uninoculated with *Pantoea* sp. (PGPR + PEG and control + PEG, respectively) (Figure 1a). Of these DEGs, 1229 genes were significantly upregulated while 854 genes were downregulated, underscoring the impact of *Pantoea* sp. on plant gene expression. Overall, these results show that *Pantoea* sp. triggered large-scale transcriptional reprogramming in *Arabidopsis* plants under drought stress. Kyoto Encyclopedia of Genes and Genomes (KEGG) analyses of the DEGs identified the different biological processes altered by *Pantoea* sp. during drought, for example, flavonoid biosynthesis, phenylpropanoid biosynthesis, carbon metabolism, and pyruvate metabolism, etc (Figure 1b). Thus, revealing the different roles of PGPR in plants under drought stress. We decided to focus on the flavonoid biosynthesis pathway because it is known to play a role in abiotic stress.^48^^-^^50^ The upregulation of the flavonoid biosynthesis pathway together with the enrichment of the phenylpropanoid biosynthesis pathway suggest that secondary metabolites are involved in PGPR-mediated drought stress tolerance. Further analyses of the DEGs showed that flavonoid and anthocyanin genes were upregulated by *Pantoea* sp. The inoculation methods included *CHS* (3.59 FC), *CHI3* (3.28 FC), MYB12 (3.4 FC), *DFRA* (3.54), *FLS2* (2.11 FC), *FLS1* (1.48 FC), and *FLS5* (1.27 FC), among others (Table 2). In order to characterize the functional classes of transcripts induced by PGPR under PEG stress, we performed GO enrichment analysis using the upregulated DEGs in the PGPR + PEG compared to the Control + PEG treatment group (Figure 1c). The enriched biological process (BP) terms were dominated by stress- and defense-related responses, including response to oxidative stress, response to water deprivation, and response to bacteria and fungi. Enriched cellular component (CC) terms highlighted secretory vesicle, plant-type cell wall, and endoplasmic reticulum lumen, among others. For molecular function (MF), the upregulated genes were enriched for redox-related activities, including peroxidase activity, oxidoreductase activity, and antioxidant activity, as well as structural constituents of the cell wall (Figure 1c). Consistent with these results, reactive oxygen species (ROS) associated biological processes were significantly overrepresented among the genes upregulated in PGPR + PEG compared with control + PEG, including response to (ROS) (GO:0000302; padj 1.38E-12) and response to oxidative stress (GO:0006979; padj 7.66E-13) (Supplementary Table 2). These terms were supported by multiple stress- and redox-associated genes, such as catalase 2 (H₂O₂ detoxification), PRXIIB (peroxiredoxin), FER1 (iron homeostasis and oxidative stress), and several heat shock proteins (HSP17.6B and HSP17.6C), which are commonly induced during oxidative challenge (Supplementary Table 2). The enriched terms further included response to hydrogen peroxide (GO:0042542; padj 4.53E-11) and cellular responses to oxidative stress (GO:0034599; padj 5.53E-05), which were supported by the induction of antioxidant and redox homeostasis genes such as CAT2 (catalase 2), peroxiredoxin IIB (PRXIIB), ascorbate peroxidase (APX), monodehydroascorbate reductase (MDAR), and glutaredoxin (GRX) (Supplementary Table 2). In addition, the response to superoxide (GO:0000303; padj 0.00418) and response to oxygen radical (GO:0000305; padj 0.00418) categories were enriched, including superoxide dismutase (SOD)-related genes [CSD1 (copper superoxide dismutase 1), FSD1/FSD3 (Fe (iron) superoxide dismutase 1/3), CCS (copper chaperone), and MSD1 (manganese superoxide dismutase 1)], indicating enhanced ROS detoxification capacity under PEG stress in PGPR-inoculated plants compared to uninoculated control (Supplementary Table 2). To confirm these results, we visualized ROS production using a DCFDA fluorescence assay. Intense, highly localized accumulation of ROS was observed in control plants, which were predominantly concentrated within the primordia of emerging lateral roots (Supplementary Figure 1). In contrast, inoculation with *Pantoea* sp. altered the plant's oxidative profile. The *Pantoea* sp. treated roots lacked the intense, localized fluorescent burst observed in the control roots but displayed a more diffuse and visually reduced DCF fluorescence across the root tip (Supplementary Figure 1). Together, these observations demonstrate that *Pantoea* sp. colonization effectively modulates the plant's localized ROS burst in roots.

**Table 2.** RNA-sequencing differentially expressed flavonoid and anthocyanins related genes.

	Log_2_-fold		
Gene ID	Change	Gene name	Gene description
AT5G15120	3.77	*PCO1*	Plant cysteine oxidase 1 [Source:UniProtKB/Swiss-Prot;Acc:Q9LXG9]
AT5G13930	3.59	*CHS*	Chalcone synthase family protein [Source:UniProtKB/TrEMBL;Acc:Q460R0]
AT4G22880	3.56	*LDOX*	AT4G22880 protein [Source:UniProtKB/TrEMBL;Acc:Q0WWD6]
AT5G42800	3.54	*DFRA*	Dihydroflavonol reductase [Source:UniProtKB/TrEMBL;Acc:B1GV15]
AT5G07990	3.52	*CYP75B1*	TT7 [Source:UniProtKB/TrEMBL;Acc:A0A178UNZ9]
AT2G47460	3.40	*MYB12*	Transcription factor MYB12 [Source:UniProtKB/Swiss-Prot;Acc:O22264]
AT5G05270	3.28	*CHI3*	Probable chalcone−flavonone isomerase 3 [Source:UniProtKB/Swiss-Prot;Acc:Q8VZW3]
AT5G17220	2.87	*GSTF12*	Glutathione S-transferase F12 [Source:UniProtKB/Swiss-Prot;Acc:Q9FE46]
AT2G44450	2.53	*BGLU15*	Beta-glucosidase 15 [Source:UniProtKB/Swiss-Prot;Acc:O64879]
AT5G63590	2.11	*FLS3*	Flavonol synthase 3 [Source:UniProtKB/Swiss-Prot;Acc:Q9FFQ5]
AT3G29590	2.05	*5MAT*	Malonyl-CoA:anthocyanidin 5-O-glucoside-6"-O-malonyltransferase [Source:UniProtKB/Swiss-Prot;Acc:Q9LJB4]
AT1G67980	1.67	*CCOAMT*	Putative caffeoyl-CoA O-methyltransferase At1g67980 [Source:UniProtKB/Swiss-Prot;Acc:Q9C9W3] Emb
AT5G58930	1.67	*-*	[Source:UniProtKB/TrEMBL;Acc:Q9FIL8]
AT5G08640	1.48	*FLS1*	Flavonol synthase/flavanone 3-hydroxylase [Source:UniProtKB/Swiss-Prot;Acc:Q96330]
AT1G64060	1.43	*RBOHF*	Respiratory burst oxidase homolog protein F [Source:UniProtKB/Swiss-Prot;Acc:O48538]
AT1G03940	1.36	*3AT1*	Coumaroyl-CoA:anthocyanidin 3-O-glucoside-6"-O-coumaroyltransferase 1 [Source:UniProtKB/Swiss-Prot;Acc:Q9ZWB4]
AT5G63600	1.27	*FLS5*	Flavonol synthase 5 [Source:UniProtKB/TrEMBL;Acc:F4KAS3]

Furthermore, our results showed there were 68 and 257 DEGs that were unique to the control + PEG and PGPR + PEG treatments respectively and 47 DEGs that were shared between both treatments (Figure 1d). Taken together, these results show that *Pantoea* sp. inoculation under PEG stress triggered a coordinated transcriptional activation of flavonoid biosynthesis together with enriched ROS detoxification pathways and ROS scavenging, providing a mechanistic basis for enhanced drought tolerance by strengthening antioxidant capacity and maintaining redox homeostasis under drought stress.

### *Pantoea* species enhances drought tolerance through the flavonoid biosynthesis pathway

**Figure 2.**
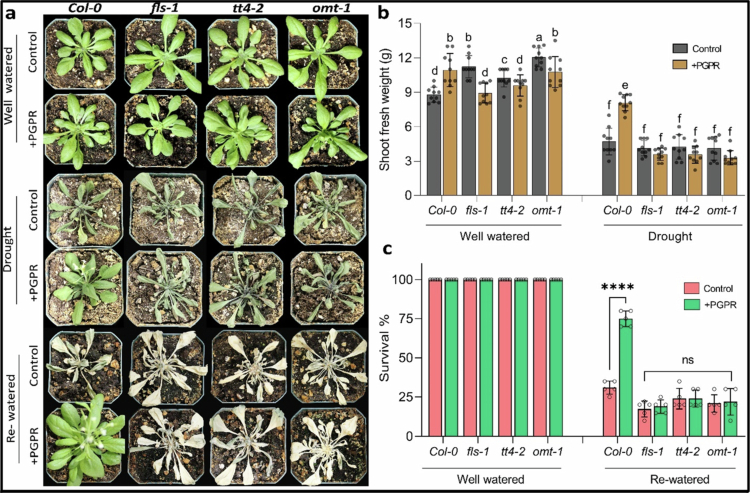
Effects of *Pantoea* species on the plant response to drought stress and rewatering: (a) representative images of plants 10 d after inoculation with PGPR under well-watered conditions, 10 d after drought stress, and 10 d after rehydration. (b) Shoot fresh weight of PGPR-inoculated and uninoculated plants under well-watered and drought stress conditions. ANOVA (Tukey's multiple comparison test), *p* < 0.05. *n* = 120. (c) Survival rates of 20-d-old *Col-0*, *fls-1*, *tt4-2*, and *omt1* plants with or without PGPR inoculation in well-watered conditions and 10 d of rewatering. ANOVA (Tukey's multiple comparison test). ****p < 0.0001 (ns = no significant difference). The experiments had two treatments (control and PGPR), two conditions (PEG and rewatering), and four plant genotypes. Each treatment had three replicates. PGPR = plant growth-promoting rhizobacteria. PEG = polyethylene glycol 6000. The PGPR used in this study is a *Pantoea* strain.

To investigate whether *Pantoea* sp. uses the flavonoid biosynthesis pathway to increase drought tolerance in plants, we carried out experiments using *Arabidopsis* wild-type Col-0 and three flavonoid biosynthesis mutants *flavonol synthase 1-3* (*fls1-3)*, *transparent testa 4-2 (tt4-2)*, and *O-methyltransferase 1* (*omt1*) in the Col-0 background.^51^ Under well-watered conditions, Col-0 plants inoculated with *Pantoea* sp. were notably larger with significantly higher shoot weight (2.2-fold) and a larger rosette diameter (1.3-fold) compared to the control (Figure 2a,b, Supplementary Figure 2a). In contrast, the noninoculated mutants exhibited higher shoot weights and larger rosette diameters compared to the *Pantoea* sp. inoculated mutants (Figure 2b, Supplementary Figure a). After withholding water for 10 d, Col-0 plants inoculated with *Pantoea* sp. appeared greener and healthier with less wilting compared to the non-inoculated plants. However, there were no differences in appearance between inoculated and noninoculated mutant plants (Figure 2a). Under drought stress, wild-type plants treated with *Pantoea* sp. had higher shoot weight and larger rosette diameter compared to the control, but no significant differences were observed between the *Pantoea* sp. treated and untreated mutants (Figure 2b, Supplementary Figure 2a). Overall, our results showed 2.3-fold and 1.3-fold increases in fresh weight and rosette diameter, respectively, in *Pantoea*-inoculated Col-0 plants under drought conditions (Figure 2b, Supplementary Figure 2a).

Chlorophyll content was measured in soil-grown wild-type and mutant plants 5 d after drought induction. Under well-watered conditions, *Pantoea*-treated wild-type plants had higher chlorophyll levels (30.8 ± 1.8 µg/g) than the uninoculated control under the same conditions (21.9 ± 1.3 µg/g). A similar trend was observed under drought stress, where *Pantoea*-inoculated Col-0 plants had a significantly higher chlorophyll content (18.9 ± 1.1 µg/g) compared to the uninoculated Col-0 plants (13.1 ± 0.8 µg/g) (Supplementary Figure 2b). The flavonoid pathway mutants had significantly higher chlorophyll content after inoculation with *Pantoea* sp. compared to the uninoculated plants under well-watered conditions, but there were no significant differences in chlorophyll content between *Pantoea*-treated and untreated mutants under drought conditions. Nonstressed plants can be ranked in the following order based on their chlorophyll content from the highest to the lowest: *Pantoea* inoculated wild-type plants, uninoculated wild-type plants, inoculated mutants and finally uninoculated mutants.

To further assess the impact of PGPR on drought tolerance, the survival rate of plants was determined under watered and drought conditions. Under well-watered conditions, a 100% survival rate was observed, with no significant difference in the survival of *Pantoea*-treated and untreated Col-0 and mutants plants (Figure 2c). *Pantoea*-inoculated Col-0 plants had a significantly higher survival rate (75.2 ± 7.1%, mean ± SD) compared to the uninoculated Col-0 plants (31 ± 5.8%, mean ± SD) under drought stress (Figure 2c). The survival rate was not significantly different between inoculated and uninoculated mutants under drought stress. These results underscore the role of *Pantoea* sp. in aiding plant tolerance to drought and indicate that a functional flavonoid biosynthesis pathway is required for *Pantoea*-induced drought tolerance.

### *Pantoea* inoculation upregulates flavonoid biosynthesis related genes under drought stress

**Figure 3.**
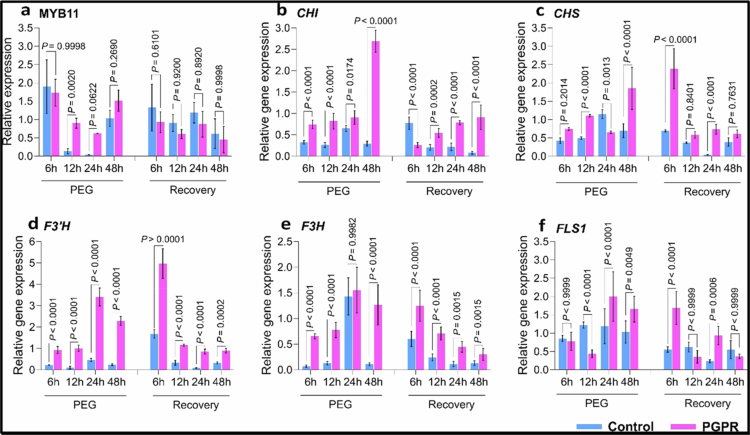
Relative expression of genes involved in the flavonoid pathway. (a) MYB11, (b) CHI, chalcone isomerase, (c) CHS, chalcone isomerase, (d) F3ʹH, flavonoid 3ʹ-hydroxylase, (e) F3H, flavanone-3-ohydroxylase, (f) FLS, flavonol synthase. ANOVA (Tukey's multiple comparison test) was used to compare gene expression levels between control and PGPR treatments at each time point. *p* < 0.05 was considered statistically significant. *n* = 120. The experiments had two treatments (control and PGPR), two conditions (PEG and rewatering), and four plant genotypes. PEG refers to the period when seedlings are transferred to MS containing 20% PEG 6000, and recovery refers to transfer from PEG media into PEGfree MS + 1.5% sucrose (recovery media). Each treatment had three replicates. PGPR = plant growth-promoting rhizobacteria. The PGPR used in this study is a *Pantoea* strain. PEG = polyethylene glycol 6000.

To validate our RNA-seq data and confirm the involvement of *Pantoea* sp. in regulating the flavonoid biosynthesis pathway during drought stress, we quantified the transcript levels of key genes in the flavonoid pathway under drought stress and recovery conditions in the absence or presence of *Pantoea* sp. (Figure 3). RT-qPCR analyses showed that *Pantoea* inoculation upregulated multiple flavonoid biosynthesis genes, such as chalcone isomerase (*CHI)*, chalcone synthase (*CHS*), flavanone 3-hydroxylase (*F3H*), flavonoid 3ʹ hydroxylase (*F3ʹH*), flavonol synthase *(FLS1*), and flavonol glycoside (MYB11) transcription factors, under PEG stress (Figure 3, Supplementary Figure 3). All of these genes were significantly upregulated in *Pantoea*-inoculated plants compared to the uninoculated control plants at 48 h after drought stress: *CHS* (*p* < 0.0001), *CHI* (*p* < 0.0001), *F3ʹH* (*p* < 0.0001), *F3H* (*p* < 0.0001), *FLS* (*p* < 0.0049), (Figure 3). However, MYB11 was significantly upregulated (*p* < 0.002) at 12 h after the onset of drought in *Pantoea*-inoculated plants but showed no significant changes at later time points (Figure 3). During the recovery phase, the gene expression patterns shifted and decreased to levels below those observed at 48 h after drought induction. At 6 h post-transfer to the recovery media, the transcript levels of *CHS* (*p* > 0.0001) and *F3H* (*p* > 0.0001) were significantly higher in *Pantoea*-inoculated plants. However, after 48 h in the recovery media, the expression levels of both genes had significantly decreased, and *CHS* expression was no longer significantly different from that in the control (*p* = 0.7631) (Figure 3). Interestingly, *CHI* exhibited a unique expression pattern, with transcript levels in *Pantoea*-inoculated Col-0 plants increasing steadily throughout the recovery phase, although the levels were significantly lower than those observed at 48 h after drought stress induction. Overall, these results indicate that *Pantoea* inoculation significantly upregulates flavonoid biosynthesis-related genes in Col-0 plants under drought stress, and gene expression decreases during the recovery phase.

### *Pantoea species* enhances flavonoid and anthocyanin accumulation under drought stress Flavonoids

**Figure 4.**
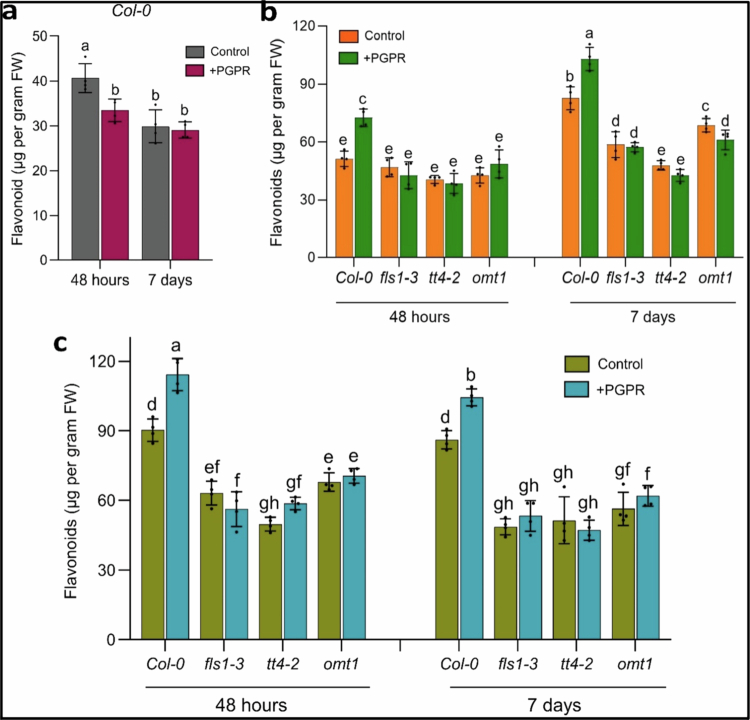
Quantification of total flavonoids in twenty-day-old seedlings of *Col-0*, *fls-1*, *tt4-2*, and *omt1* plants: (a) comparison of total flavonoids in twenty-day-old seedlings of *Col-0* plants 48 h and 7 d after inoculation with *Pantoea* species. (b) Comparison of total flavonoids in PGPR inoculated and uninoculated 20-d-old seedlings of *Col-0*, *fls-1*, *tt4-2*, and *omt1* plants 48 h and 7 d after exposure to PEG stress. (c) Comparison of total flavonoids in PGPR-inoculated and uninoculated 20-d-old seedlings of *Col-0*, *fls-1*, *tt4-2*, and *omt1* plants 48 h and 7 d after transfer to recovery media. Quercetin was used as a standard. ANOVA (Tukey's multiple comparison test), *p* < 0.05. *n* = 120. The experiment had two treatments and four plant genotypes. Each treatment had three replicates. PGPR = plant growth-promoting rhizobacteria. The PGPR used in this study is a *Pantoea* strain.

Since our RNA-seq data showed significant enrichment of the flavonoid biosynthesis pathway in the GO and KEGG terms in *Pantoea*-treated plants under drought stress (Figure 1), and our gene expression analysis showed that *Pantoea* sp. upregulated genes in the same pathway (Figure 3), we hypothesized that this bacterium enhances drought stress tolerance through the upregulation of the flavonoid pathway. First, we quantified total flavonoids in *Col-0* plants at 48 h and 7 d after inoculation with *Pantoea* sp. without exposure to drought stress (Figure 4a). At 48 h, the total flavonoid content was significantly higher in the untreated controls (40.68 ± 2.06 µg/gFW) compared to PGPR treated plants (29.90 ± 1.57 µg/gFW) (Figure 4a). However, after 7 d, the flavonoid levels had declined in the uninoculated plants (control) to the point that there were no significant differences between the control (33.47 ± 1.84 µg/gFW) and inoculated plants (PGPR) (29.09 ± 1.92 µg/gFW) treatments (Figure 4a). The quantification of flavonoids 48 h after drought stress revealed that *Pantoea*-inoculated Col-0 plants had significantly higher levels of flavonoids (72.53 ± 3.6 µg/gFW) than the uninoculated controls (51.26 ± 2.9 µg/gFW) (Figure 4b). Interestingly, the flavonoid content in the uninoculated Col-0 plants was not significantly different from the mutants 48 h after drought induction, indicating the role of *Pantoea* sp. in inducing early plant response to drought stress in the wild type plants.

At 7 d after the induction of drought stress, a marked increase in flavonoid accumulation was noted in both *Pantoea*-inoculated and uninoculated Col-0 plants (Figure 4b). By this time, the *Pantoea*-inoculated Col-0 plants accumulated significantly higher flavonoid content (103.05 ± 5.4 µg/gFW) compared to the uninoculated Col-0 plants (82.57 ± 4.4 µg/g FW) and all the flavonoid biosynthesis mutants (Figure 4b). In contrast, no significant differences were noted between the *Pantoea* inoculated or uninoculated *fls1-3*, *tt4-2*, and *omt1* mutants at 48 h or 7 d after drought induction, except for *omt1*, where *Pantoea* inoculated plants had significantly lower flavonoid contents compared to the uninoculated *omt1* mutants (Figure 4b).

During the recovery phase following drought stress, *Pantoea* inoculated Col-0 plants continued to exhibit the highest flavonoid content compared to all the other treatments, although levels decreased progressively over time. At 48 h into recovery, the PGPR-inoculated Col-0 plants had significantly higher flavonoids (114.28 ± 5.9 µg/gFW), compared to the uninoculated Col-0 plants (90.25 ± 4.8 µg/gFW) (Figure 4c). After 7 d of recovery, the flavonoid content decreased in all the plants, but the *Pantoea* sp. inoculated Col-0 plants still maintained higher levels (104.39 ± 5.6 µg/gFW) compared to the uninoculated control (86.22 ± 4.1 µg/gFW) (Figure 4c). No significant changes in flavonoid levels were observed between *Pantoea*-inoculated and uninoculated mutant plants at either 48 h or 7 d into the recovery phase (Figure 4c). Overall, the wild-type plants showed the following flavonoid pattern: no significant change in flavonoid content at 7 d after inoculation, rapid and significant increase in inoculated plants compared to control at the beginning of and during the drought, and a slower decrease in the flavonoid content of inoculated plants compared to uninoculated control plants upon rewatering.

### Anthocyanin

**Figure 5.**
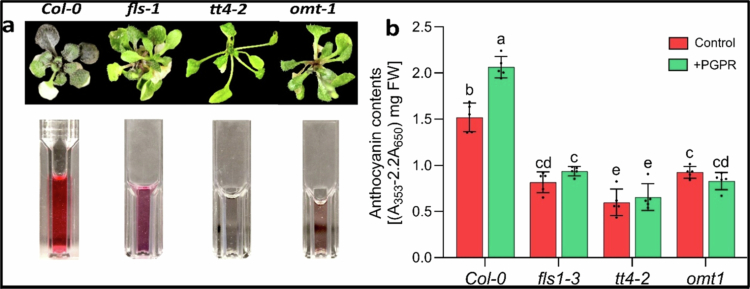
Anthocyanin content in *Arabidopsis* wild-type and flavonoid mutants under drought conditions 10 d after inoculation with *Pantoea* species: (a) representative image showing anthocyanin accumulation in 20-d-old seedlings of *Col-0*, *fls-1*, *tt4-2*, and *omt1* plants treated with *Pantoea* species for 10 d before the images were taken. (b) Quantification of anthocyanins in inoculated and uninoculated 20-d-old Col-0, *fls-1*, *tt4-2*, and *omt1* plants. ANOVA (Tukey's multiple comparison test), *p* < 0.05. *n* = 120. The experiment had two treatments and four plant genotypes. Each treatment had three replicates. PGPR = plant growth-promoting rhizobacteria. The PGPR used in this study is a *Pantoea* strain.

Quantification of total anthocyanins in *Pantoea*-inoculated and uninoculated Col-0 and flavonoid mutant plants under drought stress revealed distinct differences in accumulation levels (Figure 5a, b). Anthocyanins were detected in *Pantoea*-treated wild-type Col-0 and mutant genotypes (*fls1-3*, *tt4-2*, and *omt1*) (Figure 5a). However, *Pantoea*-inoculated wild-type plants exhibited significantly higher anthocyanin levels (2.06 ± 1.3 mg/FW) compared to the uninoculated Col-0 plants (1.51 ± 0.9 mg/FW). In contrast, no significant differences in anthocyanin levels were observed between inoculated and uninoculated mutants (Figure 5b). Among the mutants, the lowest levels were observed in *tt4-2* (Figure 5b). Our data suggest that *Pantoea-*mediated enhancement of anthocyanin accumulation may play a role in drought stress tolerance in *Arabidopsis*.

### Flavonoid pathway plays a key role in colonization of roots and *Pantoea* species attraction

**Figure 6.**
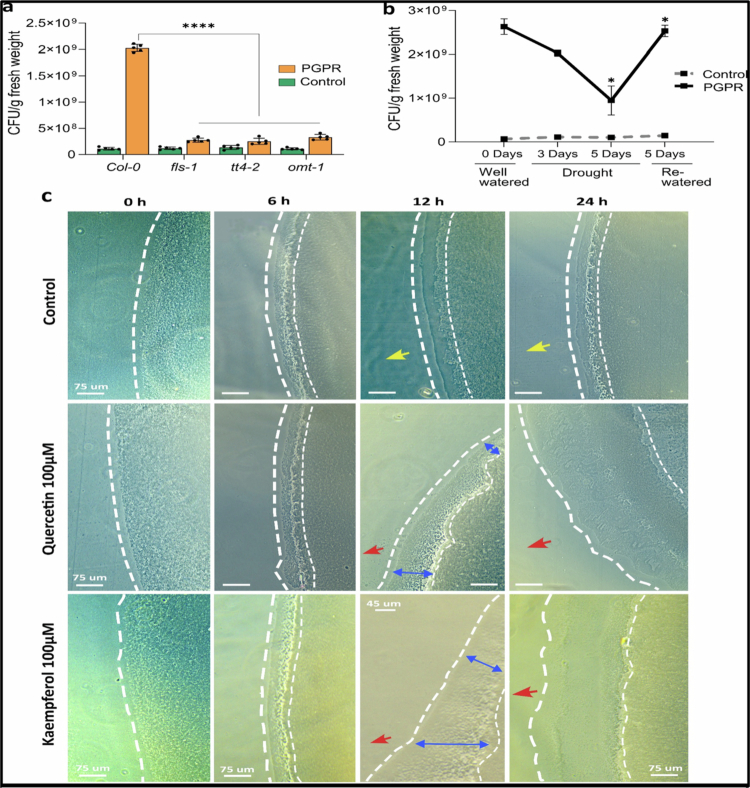
*Pantoea* species and their impact on root colonization: (a) *Pantoea* species failed to colonize the roots of the flavonoid biosynthesis pathway mutants *fls-1*, *tt4-2*, and *omt1*. Colonization was measured at 3 d after exposure to drought. ANOVA (Tukey's multiple comparison test), *p* < 0.05. (b) Drought stress led to decreased colonization of *Col-0* roots by *Pantoea* species. The asterisk indicates a significant difference relative to the preceding time point ANOVA (Tukey's test) *p* < 0.05. *n* = 120. The experiment had two treatments and four plant genotypes. Each treatment had three replicates. (c) Time-lapse phase contrast images of *Pantoea* species movement on water agar medium in the absence (control) or presence of flavonoids (quercetin and kaempferol) (100 µM) at 0, 6, 12, and 24 h. The dashed lines show the advancing bacterial swarms, while yellow and red arrows indicate the directions of the mycelial plugs with or without flavonoids, respectively. The blue arrows indicate the distance of swarming towards the flavonoid source. Scale bars, 75 μm. PGPR = plant growth-promoting rhizobacteria.

Based on our results showing that the flavonoid biosynthesis pathway mutants were not rescued by *Pantoea* sp. after drought stress (Figure 2), we investigated their root colonization by this bacterium. We observed that the *Pantoea* sp. colony forming units (CFUs) obtained from the inoculated Col-0 roots of the soil-grown plants were significantly more than those from the inoculated mutant roots and the uninoculated controls (Figure 6a). However, there were no significant differences in CFU/g between the uninoculated and inoculated mutants. Indicating that *Pantoea* sp. did not colonize mutants with a defective flavonoid pathway (Figure 6a), and a functional flavonoid pathway is essential for successful root colonization by *Pantoea* sp. The low CFUs observed in uninoculated plants could have come from the unsterile environment in which the plants were grown. Col-0 showed significantly high colonization under well-watered conditions; however, the colonization decreased with increasing drought stress (Figure 6b). The number of CFUs/g of root tissue decreased as drought progressed but increased to the original concentration upon rewatering (Figure 6b). Additionally, observations made under in vitro conditions were consistent with the soil-based results. In the sterile in vitro system, *Pantoea* robustly colonized Col-0 roots, whereas colonization in the flavonoid pathway mutants was significantly lower (Supplementary Figure 4a), further reinforcing the conclusion that an intact flavonoid pathway is required for efficient root colonization by *Pantoea*. Moreover, the temporal dynamics of colonization under PEG-induced drought stress mirrored the soil experiment, where the CFU/g were highest under nonstress conditions, declined as stress progressed, and recovered following transfer to nonstress medium (Supplementary Figure 4a).

In an assay for chemotaxis of the *Pantoea* sp., the time-lapse microscopy images showed that the bacteria exhibited a directional swarm toward quercetin and kaempferol (flavonoids) (Figure 6c). In the control, the inoculum edge remained largely stationary over 6–12 h and showed only diffuse, nonoriented spreading after 24 h, as indicated by the dashed line (Figure 6c). In contrast, in the presence of quercetin (100 µM), a leading edge emerged by 12 h and had advanced to form dense swarms oriented toward the flavonoid source after 24 h (red arrow) (Figure 6c). Similarly, a directional swarming response was also observed toward kaempferol (100 µM), with the emergence of biased migration toward the flavonoid source over time (red arrows) (Figure 6c). Collectively, these observations indicate a directional motility bias, which is consistent with chemotactic attraction.

**Figure 7.**
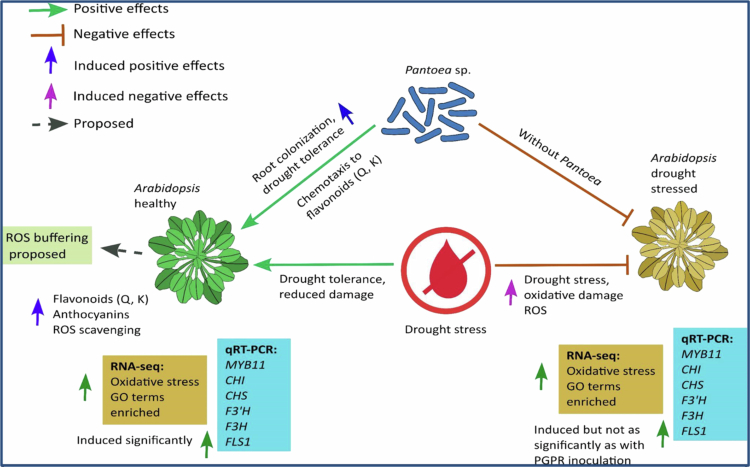
Model of *Pantoea* sp. mediated drought tolerance in *Arabidopsis*. RNA-seq analysis comparing PEG-stressed plants with or without *Pantoea* sp. (PEG + *Pantoea* vs PEG) revealed enriched gene ontology categories such as “response to oxidative stress” (GO:0006979) in *Pantoea*-inoculated plants. The model depicts *Pantoea* sp. chemotaxis toward root-exuded flavonoids [quercetin (Q) and kaempferol (K)], colonization of the rhizosphere, and induction of host stress response pathways (e.g., antioxidant defense and flavonoid/anthocyanin biosynthesis). *Pantoea* led to significant induction of genes associated with flavonoid biosynthesis in *Arabidopsis*. Further, *Pantoea* -mediated ROS buffering is proposed as a mechanism for enhancing drought tolerance in *Arabidopsis*.

In summary, our results show that *Pantoea* sp. mitigates drought stress in *Arabidopsis* by upregulating flavonoids. In addition, flavonoids act as chemoattractants to increase bacterial colonization, which enhances the protective ROS-scavenging mechanism that prevents oxidative damage and confers drought tolerance. This finding is supported by RNA-seq evidence showing enrichment of oxidative-stress–related GO terms and increases in flavonoids and anthocyanins, collectively suggesting a proposed ROS-scavenging mechanism that mitigates drought-induced oxidative damage and is summarized in Figure 7.

## Discussion

Abiotic stresses, especially climate-driven drought, compromise plant growth and yield and, consequently, environmental stability and food security.^52^ To address these challenges, novel ecologically friendly strategies, such as PGPR, are needed to increase plant stress tolerance.^53^ The beneficial effects of PGPR are more pronounced under adverse climatic conditions such as drought, making them key partners in sustainable agriculture. Numerous PGPR strains have been studied and utilized in agriculture to alleviate abiotic stress. For example, *Pseudomonas fluorescens* FY32, *Bacillus amyloliquefaciens* BA01 and *Acinetobacter calcoaceticus* AC06.^32^^,^^54^

However, the mechanisms used by many PGPR have not been fully elucidated. Our results showed that *Pantoea*-inoculated Col-0 plants had not only significantly higher biomass, rosette diameter and chlorophyll content under drought than uninoculated plants, but also were resilient upon rewatering, thus pointing to this bacterium's role in enhancing their drought tolerance and survival. Given that the frequency and severity of drought have increased in recent decades, plants are likely to experience multiple droughts throughout their lifetime, and their resilience after water shortages is crucial to their survival.^55^ Therefore, understanding the mechanism used by PGPR to mitigate drought stress in plants is both important and timely.

Significant accumulation of flavonoids in inoculated wild type and not in inoculated flavonoid mutants (*fls1-3*, *tt4-2*, and *omt1*) under drought and failure of *Pantoea* sp. to rescue these mutants after rewatering indicated that a functional flavonoid pathway is required for *Pantoea-*induced drought tolerance in plants. Plants naturally accumulate flavonoids in response to various abiotic stresses, such as drought, heat, and UV, etc.^56^^,^^57^ Flavonoids are well-known secondary metabolites with antioxidant properties that are capable of scavenging ROS that are produced in much higher concentrations during abiotic stress.^58^ If the significant amounts of ROS produced under abiotic stress (e.g., drought) are not properly countered, they can jeopardize plant survival because they lead to cell death and tissue necrosis.^59^ In this study, the higher survival rate observed in the inoculated plants after drought stress can therefore be attributed in part to enhanced flavonoid production as a result of *Pantoea* inoculation, which enhanced the ability of the plants to scavenge ROS during drought stress. Across the PEG stressed plants, our data shows that *Pantoea* inoculation shifted the transcriptome toward oxidative stress defense, where BP terms (ROS, H₂O₂, and superoxide) and MF terms (oxidoreductase, peroxidase, and antioxidant activity) were among the most significantly enriched. This antioxidant-based transcriptional reprogramming is consistent with drought literature showing that PGPR commonly enhances ROS scavenging and redox buffering capacity across different plants, which is often linked to improved stress acclimation.^33^ It also fits the broader priming framework, where beneficial microbes can prime and precondition plants for a faster and stronger activation of antioxidant capacity to reduce oxidative injury under water deficit.^60^ Therefore, we can deduce that ROS detoxification may represent one component of the *Pantoea*-induced flavonoid-mediated drought tolerance observed here. The observed reduction in flavonoid levels during the recovery phase aligns with previous reports that showed a decline in flavonoids upon rewatering of drought-stressed plants.^48^^,^^61^ This study shows that *Pantoea-*mediated drought tolerance in *Arabidopsis* involves the flavonoid biosynthesis pathway, as demonstrated by the accumulation of flavonoids and the upregulation of genes in the flavonoid pathway under drought stress in the wild type but not in the mutants. Consequently, *Pantoea* inoculation only conferred drought tolerance in wild-type plants but not in flavonoid-deficient mutants. Thus, proving that our hypothesis (*Pantoea* confers drought tolerance in plants by upregulating the flavonoid biosynthesis pathway) was true.

The flavonoid biosynthesis mutants did not exhibit increased flavonoid accumulation upon *Pantoea* inoculation, except for the partial increase observed in *omt1-*and ultimately, the mutants had significantly lower survival rates during drought stress compared to the wild type. The *fls-1* mutant is compromised in flavonoid biosynthesis but can still produce some intermediate products in the pathway.^51^ The *omt1* mutant is able to synthesize quercetin and kaempferol in shoot and root tissues, whereas the *tt4-2* mutant synthesizes no flavonoids.^51^ The ability of *omt1* to synthesize limited amounts of flavonoids may explain its significantly larger rosette diameter under drought conditions after *Pantoea* inoculation, as well as its higher flavonoid concentrations compared to the other mutants. This intermediate phenotype is likely as a result of the specific enzymatic deficiency in *omt1* that affect O-methyltransferase activity involved in the methylation of certain flavonols, but does not fully abolish the production of all flavonoids.^51^ Therefore, *omt1* plants can still synthesize reduced levels of some key flavonoid intermediates, such as quercetin and kaempferol, but in contrast, mutants with complete blocks in flavonoid biosynthesis (e.g. *tt4-2*) did not show this intermediate response.^51^^,^^62^ However, the residual flavonoids in *omt1* were not enough for sufficient root colonization, increment in shoot weight and for *Pantoea-*induced drought tolerance. The fact that none of the flavonoid mutants showed enhanced root colonization and improved drought stress survival demonstrates a strong association between *Pantoea*-induced flavonoid accumulation and improved drought tolerance in *Arabidopsis* plants. The inability of the flavonoid-deficient mutant *tt4-2* to increase the concentration of flavonoids in both inoculated and uninoculated plants under drought stress and its significantly lower survival rate under drought compared to inoculated wild-type plants suggest that flavonoids may directly contribute to the drought resilience conferred by *Pantoea* sp. However, because the tt4 mutation can have pleiotropic effects on plant physiology,^63^ we used other flavonoid biosynthesis mutants that showed similar phenotypes. Nevertheless, we acknowledge that these results are largely correlational relationship and do not conclusively prove causality. Therefore, further experiments (e.g., exogenous application of flavonoids to the mutants) would be needed to definitively establish a causal relationship. Our findings align with previous reports that show that the overaccumulation of antioxidant flavonoids (including anthocyanins) can increase plant drought tolerance.^18^

Given that, *Pantoea* inoculation did not cause the accumulation of flavonoids in Col-0 plants before drought stress induction and that, upon initiating drought stress, the flavonoid content rapidly and significantly increased in inoculated wild-type plants compared to the uninoculated control, combined with the fact that the primed plants maintained the higher flavonoid concentrations for a longer time and had a significantly higher survival rate than the uninoculated under drought conditions, which points to the priming of the plants by the bacterium. This response is characteristic of stress priming, in which root colonization by PGPR (*Pantoea* sp. in this case) elicits a primed state that does not involve a strong defense response from the *Arabidopsis* plants but allows faster and more sustained induction of specific stress response pathways upon subsequent exposure to stress.^64^ The failure to increase flavonoids without drought stress is exactly what is expected in priming, which is an adaptive, low-cost strategy in plants observed under induced systemic resistance/systemic acquired resistance and abiotic-stress memory.^64^^,^^65^

In this study, the anthocyanin content significantly increased in the inoculated wild-type plants but not in the mutants. Anthocyanins are synthesized downstream of the flavonoid biosynthesis pathway and play crucial roles in plant responses such as drought stress tolerance.^66^ The increased accumulation of flavonoids and anthocyanins in *Pantoea*-inoculated wild-type plants is supported by the upregulation of key flavonoid biosynthetic genes *CHI*, *CHS*, *F3ʹH*, *F3H*, *FLS*, and MYB11, and genes involved in anthocyanin production (*DFR* and *ANS*) have shown that *Pantoea-*induced flavonoid and anthocyanin biosynthesis are involved in mediating drought stress tolerance. Anthocyanins are known to exhibit antioxidant activity that mitigates the oxidative damage that results from ROS accumulation under abiotic stress in plants.^18^^,^^48^ This antioxidant function of anthocyanins protects cellular components and is one mechanism by which flavonoid pathway upregulation can improve drought tolerance. While these findings strongly point to increased accumulation of anthocyanin and flavonoid as playing significant roles in mitigating drought stress, targeted metabolomics analysis (e.g., LC–MS) to identify and quantify specific compounds are planned in our future studies. Such analyses provide definitive evidence linking gene expression to metabolite accumulation and further clarify the mechanistic basis of drought tolerance.

Drought stress typically triggers a significant reduction in chlorophyll content due to chlorophyll degradation, as observed in our study and in other experiments.^67^ However, *Pantoea* inoculated Col-0 plants exhibited higher chlorophyll content than the uninoculated controls indicating that inoculated plants maintained a more efficient photosynthetic capacity under drought stress. Other beneficial bacteria have been shown to enhance plant performance under drought conditions by enhancing photosynthesis efficiency and increasing chlorophyll content, thereby sustaining photosynthesis and survival during drought.^26^^,^^68^ These significant differences in chlorophyll content between the inoculated and uninoculated plants under drought stress were observed only in wild-type Col-0 plants but not in flavonoid mutants. These results underscore the importance of the flavonoid biosynthesis pathway in enhancing chlorophyll retention under drought stress conditions.^18^^,^^69^

Root colonization is a prerequisite for PGPR to exert their beneficial effects on plants.^33^^,^^70^ Successful colonization is influenced by host factors such as root exudates and PGPR traits such as motility, adhesion to the root, and growth rate, etc.^70^ In the present study, we observed that root colonization in inoculated Col-0 plants was significantly higher than that in the mutants, thus indicating that a functional flavonoid biosynthesis pathway is necessary for effective colonization of *Arabidopsis* roots by *Pantoea* sp. In addition, our chemotaxis assay showed that the *Pantoea* strain used in this study was attracted to flavonoids. These results are consistent with previous reports which have shown that flavonoids might act directly as chemoattractants. For example, *Rhizobium leguminosarum* was reported to migrate toward certain flavonoid nod-gene inducers (e.g. apigenin, luteolin) in vitro.^71^

Collectively, our results show that flavonoids play a vital role in attracting the *Pantoea* sp. strain used in this study to host roots. This is the first report of *Pantoea* sp. being attracted to host roots by flavonoids. Although the reason behind the lack of colonization in the mutants was not investigated, we presume that it could be due to many factors since the mutations could have caused pleiotropic effects, such as altered composition of root exudates leading to loss of metabolites required for bacterial attraction or attachment.^72^^,^^73^ Effective root colonization allows PGPR to establish biochemical and physical interactions that are essential for promoting plant growth, inducing defense, modulating physiological processes and ultimately leading to systemic resilience and abiotic stress tolerance.^74^^-^^76^ The mutants used in the study did not reap the benefits of the *Pantoea* species*-*induced drought tolerance due to the inability of the bacterium to colonize their roots. Therefore, optimizing the process through which *Pantoea* sp. successfully establish effective root colonization is pivotal for maximizing its beneficial effects. The stable *Pantoea* sp. colonization observed in Col-0 under nonstress conditions and the sustained recovery following drought stress highlights the robustness of *Pantoea* species–*Arabidopsis* interactions. This stability is integral to establishment of the symbiotic interactions that are vital for PGPR enhanced nutrient availability and protection from abiotic stress.^77^^,^^78^

These results collectively support a model in which PGPR inoculation stimulates flavonoid biosynthesis, thereby enhancing the plant's ability to scavenge reactive oxygen species, maintaining metabolic homeostasis, stabilizing cellular processes, and accelerating poststress recovery rates. This is due to the activity of flavonoids, which act as potent modulators and antioxidants of stress signaling, mitigating the oxidative damage that typically accompanies osmotic and drought stress.^79^ Additionally, the ability of flavonoids to regulate stress-related genes and scavenge free radicals leads to enhanced plant tolerance to drought stress.^60^^,^^80^ Flavonoids have also been shown to enhance tolerance against multiple abiotic stressors, including salinity, UV radiation, drought, cold and heavy metals.^81^^,^^82^ Given that treatment with *Pantoea* sp. elevates the flavonoid content and increased flavonoid levels under saline conditions enhance tissue tolerance and sodium ion pumping rates,^83^ the *Pantoea*-induced flavonoids can contribute to both salt and drought tolerance.

This work shows that PGPR can significantly enhance drought tolerance through different mechanisms that involve alterations at the molecular, biochemical and physiological levels. Furthermore, *Pantoea* inoculation significantly altered *Arabidopsis* transcription under PEG-induced drought stress, activating pathways associated with plant metabolism, secondary metabolite production and stress signalling. We demonstrated that flavonoids are essential for root colonization and PGPR-induced drought tolerance in *Arabidopsis*. This observation underscores the novelty of this study because it is the first time *Pantoea* sp. and PGPR in general have been shown to induce drought tolerance through the upregulation of the flavonoid biosynthesis pathway. While this study was conducted under controlled conditions, similar effects are expected in field crops because of the conserved mechanisms of plant–PGPR interactions.^33^ However, the outcome may be modified by the host interaction with the native microbial population and by weather variability. Given their importance, field trials are planned in the future to demonstrate the translational efficacy of *Pantoea* sp. in enhancing drought resilience in agronomically important crops.

## Conclusions

Overall, our findings demonstrate that *Pantoea* sp., a PGPR, enhances drought tolerance in *Arabidopsis* by regulating both plant metabolism and physiology. Transcriptomic analyses and biochemical assays showed that treatment with *Pantoea* sp. induces flavonoid and anthocyanin production. This new insight contributes to our knowledge base in the development of bacterial inoculants that improve crop tolerance to drought stress. The application of PGPR to mitigate abiotic stress is an ecologically sustainable approach because it harnesses plant‒microbe associations in an environmentally friendly manner. As drought episodes increase across the globe, PGPR-based strategies coupled with molecular breeding for increased flavonoid biosynthesis could significantly contribute to improving and maintaining crop yields. Despite numerous studies showing the efficacy of PGPR inoculation under laboratory conditions, there is limited evidence of PGPR success under field conditions during drought. Further, developing viable PGPR inoculants is challenged by strain specificity, environmental variability, and competition with native microbiota, which can limit their efficacy outside of laboratory conditions. Consequently, elucidation of the mechanisms employed by PGPR for drought stress tolerance and recovery under field conditions, in addition to advances in formulation and strain selection, will lead to the development of resilient crop production systems. The knowledge gained from this and other similar studies can be harnessed in the design of next-generation synthetic communities tailored for drought-prone areas. Based on the results obtained in this study, future studies will focus on (i) development and optimization of inoculant formulations for field application, (ii) elucidating the molecular cross talk between PGPR signals, and hormone networks in plants.

## Supplementary Material

Supplementary material Supplementary material

## References

[1] Food and Agriculture Organization of the United Nations (FAO). (2021). The State of Food and Agriculture 2021: Making agrifood systems more resilient to shocks and stresses. Rome: FAO. doi:10.4060/cb4476en.

[2] Venkatappa M, Sasaki N, Han P, Abe I. Impacts of droughts and floods on croplands and crop production in Southeast Asia - an application of Google Earth Engine. Sci Total Environ. 2021;795:148829148829. doi: 10.1016/j.scitotenv.2021.148829. 34252779

[3] Yin J, Gentine P, Slater L, Gu L, Pokhrel Y, Hanasaki N, Guo S, Xiong L, Schlenker W. Future socio-ecosystem productivity threatened by compound Drought–heatwave events. Nat Sustain. 2023;6(3):259–272. doi: 10.1038/s41893-022-01024-1.

[4] Jansson JK, Hofmockel KS. Soil microbiomes and climate change. Nat Rev Microbiol. 2020;18(1):35–46. 31586158 10.1038/s41579-019-0265-7

[5] Chen L, Brun P, Buri P, Fatichi S, Gessler A, McCarthy MJ, Pellicciotti F, Stocker B, Karger DN. Global increase in the occurrence and impact of multiyear droughts. Science. 2025;387(6731):278–284. doi: 10.1126/science.ado4245. 39818908

[6] Qing Y, Wang S, Ancell BC, Yang Z-L. Accelerating flash droughts induced by the joint influence of soil moisture depletion and atmospheric aridity. Nat Commun. 2022;13(1):1139. doi: 10.1038/s41467-022-28752-4. 35241658 PMC8894453

[7] Suarez-Gutierrez L, Müller WA, Marotzke J. Extreme heat and drought typical of an end-of-century climate could occur over Europe soon and repeatedly. Commun Earth Environ. 2023;4(1):415. doi: 10.1038/s43247-023-01075-y.

[8] Haghpanah M, Hashemipetroudi S, Arzani A, Araniti F. Drought tolerance in plants: physiological and molecular responses. Plants. 2024;13(21):2962. doi: 10.3390/plants13212962. 39519881 PMC11548289

[9] Wang L, Chen M, Lam P-Y, Dini-Andreote F, Dai L, Wei Z. Multifaceted roles of flavonoids mediating plant-microbe interactions. Microbiome. 2022;10(1):233. doi: 10.1186/s40168-022-01420-x. 36527160 PMC9756786

[10] Alseekh S, Perez de Souza L, Benina M, Fernie AR. The style and substance of plant flavonoid decoration; towards defining both structure and function. Phytochemistry. 2020;174:112347112347. doi: 10.1016/j.phytochem.2020.112347. 32203741

[11] Rowan DD, Cao M, Lin-Wang K, Cooney JM, Jensen DJ, Austin PT, Hunt MB, Lin‐Wang K, Norling C, Hellens RP, et al. Environmental regulation of leaf colour in red 35S:PAP1 *Arabidopsis thaliana*. New Phytol. 2009;182(1):102–115. doi: 10.1111/j.1469-8137.2008.02737.x. 19192188

[12] Stracke R, Favory J-J, Gruber H, Bartelniewoehner L, Bartels S, Binkert M, Funk M, Weisshaar B, Ulm R. The *Arabidopsis* bZIP transcription factor HY5 regulates expression of the PFG1/MYB12 gene in response to light and Ultraviolet-B radiation. Plant Cell Environ. 2010;33(1):88–103. 19895401 10.1111/j.1365-3040.2009.02061.x

[13] Tohge T, Matsui K, Ohme-Takagi M, Yamazaki M, Saito K. Enhanced radical scavenging activity of genetically modified *Arabidopsis* seeds. Biotechnol Lett. 2005;27(5):297–303. doi: 10.1007/s10529-005-0683-7. 15834789

[14] Mathesius U. The role of the flavonoid pathway in *Medicago truncatula* in root nodule formation. A review. In de Bruijn FJ (Ed.), The Model Legume Medicago Truncatula. 2020. pp. 434–438. Hoboken, NJ: John Wiley & Sons. doi: 10.1002/9781119409144.ch54.

[15] Zhang J, Subramanian S, Stacey G, Yu O. Flavones and flavonols play distinct critical roles during nodulation of medicago truncatula by *Sinorhizobium meliloti*. Plant J Cell Mol Biol. 2009;57(1):171–183. doi: 10.1111/j.1365-313X.2008.03676.x. 18786000

[16] Nazari F, Safaie N, Soltani BM, Shams-Bakhsh M, Sharifi M. Bacillus subtilis affects miRNAs and flavanoids production in agrobacterium-tobacco interaction. Plant Physiol Biochem. 2017;118(September):98–106. doi: 10.1016/j.plaphy.2017.06.010. 28624685

[17] Mekureyaw MF, Pandey C, Hennessy RC, Nicolaisen MH, Liu F, Nybroe O, Roitsch T. The cytokinin-producing plant beneficial bacterium Pseudomonas fluorescens G20-18 primes tomato (Solanum lycopersicum) for enhanced drought stress responses. J Plant Physiol. 2022;270:153629. doi: 10.1016/j.jplph.2022.153629. 35151004

[18] Nakabayashi R, Mori T, Saito K. Alternation of flavonoid accumulation under drought stress in *Arabidopsis thaliana*. Plant Signal Behav. 2014;9(8):e29518. doi: 10.4161/psb.29518. 25763629 PMC4203635

[19] Mousa WK, Shearer C, Limay-Rios V, Ettinger CL, Eisen JA, Raizada MN. Root-hair endophyte stacking in finger millet creates a physicochemical barrier to trap the fungal pathogen *Fusarium graminearum*. Nat Microbiol. 2016;1(12):16167. doi: 10.1038/nmicrobiol.2016.167. 27669453

[20] Liu H, Li J, Singh BK. Harnessing co-evolutionary interactions between plants and *Streptomyces* to combat drought stress. Nat Plants. 2024;10(8):1159–1171. doi: 10.1038/s41477-024-01749-1. 39048724

[21] Beneduzi A, Ambrosini A, Passaglia LMP. Plant growth-promoting rhizobacteria (PGPR): their potential as antagonists and biocontrol agents. Genet Mol Biol. 2012;35(4 (suppl):1044–1051. doi: 10.1590/S1415-47572012000600020. 23411488 PMC3571425

[22] Moore JAM, Abraham PE, Michener JK, Muchero W, Cregger MA. Ecosystem consequences of introducing plant growth promoting rhizobacteria to managed systems and potential legacy effects. New Phytol. 2022;234(6):1914–1918. doi: 10.1111/nph.18010. 35098533 PMC9314638

[23] Sonbarse PP, Kiran K, Sharma P, Parvatam G. Biochemical and molecular insights of PGPR application for the augmentation of carotenoids, tocopherols, and folate in the foliage of *Moringa oleifera*. Phytochemistry. 2020;179:112506112506. doi: 10.1016/j.phytochem.2020.112506. 32920264

[24] Tang A, Haruna AO, Majid NMA, Jalloh MB. Potential PGPR properties of cellulolytic, nitrogen-fixing, phosphate-solubilizing bacteria in rehabilitated tropical forest soil. Microorganisms. 2020;8(3):442. doi: 10.3390/microorganisms8030442. 32245141 PMC7143980

[25] Coban O, De Deyn GB, van der Ploeg M. Soil microbiota as game-changers in restoration of degraded lands. Science. 2022;375 e0725. 10.1126/science.abe0725 35239372

[26] Batool T, Ali S, Seleiman MF, Naveed NH, Ali A, Ahmed K, Abid M, Rizwan M, Shahid MR, Alotaibi M, et al. Plant growth promoting rhizobacteria alleviates drought stress in potato in response to suppressive oxidative stress and antioxidant enzymes activities. Sci Rep. 2020;10(1):16975. doi: 10.1038/s41598-020-73489-z. 33046721 PMC7550571

[27] Chieb M, Gachomo EW. The role of plant growth promoting rhizobacteria in plant drought stress responses. BMC Plant Biol. 2023;23(1):407. doi: 10.1186/s12870-023-04403-8. 37626328 PMC10464363

[28] Abd El-Daim IA, Bejai S, Meijer J. Bacillus velezensis 5113 induced metabolic and molecular reprogramming during abiotic stress tolerance in wheat. Sci Rep. 2019;9(1):16282. doi: 10.1038/s41598-019-52567-x. 31704956 PMC6841942

[29] Singh RP, Jha PN. The multifarious PGPR *Serratia marcescens* CDP-13 augments induced systemic resistance and enhanced salinity tolerance of wheat (*Triticum aestivum* L.). PLoS One. 2016;11(6):e0155026. doi: 10.1371/journal.pone.0155026. 27322827 PMC4913913

[30] Porcel R, Zamarreño ÁM, García-Mina JM, Aroca R. Involvement of plant endogenous ABA in bacillus megaterium PGPR activity in tomato plants. BMC Plant Biol. 2014;14(January):36. doi: 10.1186/1471-2229-14-36. 24460926 PMC3903769

[31] Khoso M, Ahmed S, Wagan I, Alam A, Hussain Q, Ali S, Saha TR, Poudel H, Manghwar F, Liu. Impact of plant growth-promoting rhizobacteria (PGPR) on plant nutrition and root characteristics: current perspective. Plant Stress. 2024;11:100341100341. doi: 10.1016/j.stress.2023.100341.

[32] Eswaran SU, Devi L, Sundaram K, Perveen NA, Bukhari RZ, Sayyed. Osmolyte-producing microbial biostimulants regulate the growth of *Arachis hypogaea* L. Under drought stress. BMC Microbiol. 2024;24(1):165. doi: 10.1186/s12866-024-03320-6. 38745279 PMC11094965

[33] Vries FT, de RI, Griffiths CG, Knight O, Nicolitch A, Williams. Harnessing rhizosphere microbiomes for drought-resilient crop production. Science. 2020;368(6488):270–274. doi: 10.1126/science.aaz5192. 32299947

[34] Chapman JM, Muday GK. Flavonols modulate lateral root emergence by scavenging reactive oxygen species in *Arabidopsis thaliana*. J Biol Chem. 2021;296:100222100222. doi: 10.1074/jbc.RA120.014543. 33839683 PMC7948594

[35] Duarte G, Turqueto PK, Pandey N, Vaid S, Alseekh AR, Fernie Z, Nikoloski RAE, Laitinen. Plasticity of rosette size in response to nitrogen availability is controlled by an RCC1-family protein. Plant Cell Environ. 2021;44(10):3398–3411. doi: 10.1111/pce.14146. 34228823

[36] Nam HI, Shahzad Z, Dorone Y, Clowez S, Zhao K, Bouain N, Cho H, Rhee SY, Rouached H. Interdependent iron and phosphorus availability controls photosynthesis through retrograde signaling. bioRxiv. 2021. 10.1101/2021.02.11.430802. PMC8664907 34893639

[37] Patro R, Duggal G, Love MI, Irizarry RA, Kingsford C. Salmon provides fast and bias-aware quantification of transcript expression. Nat Methods. 2017;14(4):417–419. doi: 10.1038/nmeth.4197. 28263959 PMC5600148

[38] Calixto CPG, Guo W, James AB, Tzioutziou NA, Entizne JC, Panter PE, Knight H, Nimmo HG, Zhang R, Brown JWS. Rapid and Dynamic Alternative Splicing Impacts the Arabidopsis Cold Response Transcriptome. The Plant Cell. 2018;30(7):1424–1444. doi: 10.1105/tpc.18.00177. 29764987 PMC6096597

[39] Guo W, Tzioutziou NA, Stephen G, Milne I, Calixto CPG, Waugh R, Brown JWS, Zhang R. 3D RNA-seq: a powerful and flexible tool for rapid and accurate differential expression and alternative splicing analysis of RNA-seq data for biologists. RNA Biology. 2020;18(11):1574–1587. doi: 10.1080/15476286.2020.1858253. 33345702 PMC8594885

[40] R Core Team. (2020) R: A language and environment for statistical computing. Vienna, Austria: R Foundation for Statistical Computing.

[41] Law Charity W, Chen Yunshun, Shi Wei, Smyth Gordon K. voom: Precision weights unlock linear model analysis tools for RNA-seq read counts. Genome Biol. 2014;15(2), R29. doi: 10.1186/gb-2014-15-2-r29. 24485249 PMC4053721

[42] Ritchie ME, Phipson B, Wu D, Hu Y, Law CW, Shi W, Smyth GK. limma powers differential expression analyses for RNA-sequencing and microarray studies. Nucleic Acids Res. 2015;43(7):e47. doi: 10.1093/nar/gkv007. 25605792 PMC4402510

[43] Ashburner M, Ball CA, Blake JA, Botstein D, Butler H, Cherry JM, Davis AP, Dolinski K, Dwight SS, Eppig JT, et al. Gene ontology: tool for the unification of biology. Nat Genet. 2000;25(1):25–29. doi: 10.1038/75556. 10802651 PMC3037419

[44] Kanehisa M, Goto S. KEGG: Kyoto Encyclopedia of Genes and Genomes. Nucleic Acids Res. 2000;28(1):27–30. doi: 10.1093/nar/28.1.27. 10592173 PMC102409

[45] Sharma A, Badola PK, Bhatia C, Sharma D, Trivedi PK. Primary transcript of miR858 encodes regulatory peptide and controls flavonoid biosynthesis and development in *Arabidopsis*. Nat Plants. 2020;6(10):1262–1274. doi: 10.1038/s41477-020-00769-x. 32958895

[46] Li S, Wang W, Gao J, Yin K, Wang R, Wang C, Petersen M, Mundy J, Qiu J-L. MYB75 phosphorylation by MPK4 is required for light-induced anthocyanin accumulation in *Arabidopsis*. Plant Cell. 2016;28(11):2866–2883. doi: 10.1105/tpc.16.00130. 27811015 PMC5155340

[47] Ye X, Li Z, Luo X, Wang W, Li Y, Li R, Zhang B, Qiao Y, Zhou J, Fan J, et al. A predatory myxobacterium controls cucumber fusarium wilt by regulating the soil microbial community. Microbiome. 2020;8(1):49. doi: 10.1186/s40168-020-00824-x. 32252828 PMC7137222

[48] Nakabayashi R, Yonekura-Sakakibara K, Urano K, Suzuki M, Yamada Y, Nishizawa T, Matsuda F, Yonekura‐Sakakibara K, Kojima M, Sakakibara H, et al. Enhancement of oxidative and drought tolerance in *Arabidopsis* by overaccumulation of antioxidant flavonoids. Plant J Cell Mol Biol. 2014;77(3):367–379. doi: 10.1111/tpj.12388. PMC4282528 24274116

[49] Tohge T, de Souza LP, Fernie AR. Current understanding of the pathways of flavonoid biosynthesis in model and crop plants. J Exp Bot. 2017;68:4013–4028. doi: 10.1093/jxb/erx177. 28922752

[50] Li B, Fan R, Fan Y, Liu R, Zhang H, Chen T, Liu J, Li H, Zhao X, Song C-P. The flavonoid biosynthesis regulator PFG3 confers drought stress tolerance in plants by promoting flavonoid accumulation. Environ Exp Bot. 2022;196:104792. doi: 10.1016/j.envexpbot.2022.104792.

[51] Gayomba SR, Muday GK. Flavonols regulate root hair development by modulating accumulation of reactive oxygen species in the root epidermis. Development. 2020;147(8):dev185819. doi: 10.1242/dev.185819. 32179566

[52] Balting DF, AghaKouchak A, Lohmann G, Ionita M. Northern hemisphere drought risk in a warming climate. npj Clim Atmos Sci. 2021;4(1):61. doi: 10.1038/s41612-021-00218-2.

[53] Bandopadhyay S, Li X, Bowsher AW, Last RL, Shade A. Disentangling Plant- and environment-mediated drivers of active rhizosphere bacterial community dynamics during short-term drought. Nat Commun. 2024;15(1):6347. doi: 10.1038/s41467-024-50463-1. 39068162 PMC11283566

[54] Kazemi Oskuei B, Bandehagh A, Farajzadeh D, Asgari Lajayer B, Shu W, Astatkie T. Effects of *Pseudomonas Fluorescens* FY32 on canola (*Brassica napus* L.) cultivars under drought stress induced by polyethylene glycol. J Crop Health. 2024;76(1):251–260. doi: 10.1007/s10343-023-00958-6.

[55] Mishra AK, Singh VP. A review of drought concepts. J Hydrol. 2010;391(1–2):202–216. doi: 10.1016/j.jhydrol.2010.07.012.

[56] Rao M, Junaid Y, Xu X, Tang Y, Huang J, Liu X, Deng Q, Xu. CsCYT75B1, a citrus CYTOCHROME P450 gene, is involved in accumulation of antioxidant flavonoids and induces drought tolerance in transgenic *Arabidopsis*. Antioxidants. 2020;9(2):161. doi: 10.3390/antiox9020161. 32079281 PMC7070963

[57] Sun J, Qiu C, Ding Y, Wang Y, Sun L, Fan K, Gai Z, Dong G, Li X, Song L. Fulvic acid ameliorates drought stress-induced damage in tea plants by regulating the ascorbate metabolism and flavonoids biosynthesis. BMC Genom. 2020;21(1):411. doi: 10.1186/s12864-020-06815-4. PMC7301537 32552744

[58] Di Ferdinando M, Brunetti C, Fini A, Tattini M. Flavonoids as antioxidants in plants under abiotic stresses. In Ahmad P, Prasad MNV (Eds.), Abiotic Stress Responses in Plants: Metabolism, Productivity and Sustainability. 2012. pp. 159–179. New York, NY: Springer New York. doi: 10.1007/978-1-4614-0634-1_9.

[59] Petrov V, Hille J, Mueller-Roeber B, Gechev TS. ROS-mediated abiotic stress-induced programmed cell death in plants. Front Plant Sci. 2015;6:69. doi: 10.3389/fpls.2015.00069. 25741354 PMC4332301

[60] Li C, Qiu X, Hou X, Li D, Jiang M, Cui X, Pan X, Shao F, Xie D, Chiang VL, et al. Polymerization of proanthocyanidins under the catalysis of miR397a-regulated laccases in *Salvia miltiorrhiza* and *Populus trichocarpa*. Nat Commun. 2025;16(1):1513. doi: 10.1038/s41467-025-56864-0. 39929881 PMC11811200

[61] Buer CS, Imin N, Djordjevic MA. Flavonoids: new roles for old molecules. J Integr Plant Biol. 2010;52(1):98–111. doi: 10.1111/j.1744-7909.2010.00905.x. 20074144

[62] Daryanavard H, Postiglione AE, Mühlemann JK, Muday GK. Flavonols modulate plant development, signaling, and stress responses. Curr Opin Plant Biol. 2023;72:102350102350. doi: 10.1016/j.pbi.2023.102350. 36870100 PMC10372886

[63] Buer CS, Muday GK. The transparent testa4 mutation prevents flavonoid synthesis and alters auxin transport and the response of *Arabidopsis* roots to gravity and light. Plant Cell. 2004;16(5):1191–1205. doi: 10.1105/tpc.020313. 15100399 PMC423209

[64] Conrath U, Beckers GJM, Langenbach CJG, Jaskiewicz MR. Priming for enhanced defense. Annu Rev Phytopathol. 2015;53(1):97–119. doi: 10.1146/annurev-phyto-080614-120132. 26070330

[65] Mauch-Mani B, Baccelli I, Luna E, Flors V. Defense priming: an adaptive part of induced resistance. Annu Rev Plant Biol. 2017;68(1):485–512. doi: 10.1146/annurev-arplant-042916-041132. 28226238

[66] Jiang N, Grotewold E. Flavonoids and derived anthocyanin pigments in plants-structure, distribution, function, and methods for quantification and characterization. Cold Spring Harb Protoc. August. 2024. doi: 10.1101/pdb.top108516. 39187304

[67] Hu F, Zhang Y, Guo J. Effects of drought stress on photosynthetic physiological characteristics, leaf microstructure, and related gene expression of yellow horn. Plant Signal Behav. 2023;18(1):2215025. doi: 10.1080/15592324.2023.2215025. 37243677 PMC10228403

[68] Yang N, Nesme J, Røder HL, Li X, Zuo Z, Petersen M, Burmølle M, Sørensen SJ. Emergent bacterial community properties induce enhanced drought tolerance in *Arabidopsis*. NPJ Biofilms Microbiomes. 2021;7(1):82. doi: 10.1038/s41522-021-00253-0. 34795326 PMC8602335

[69] Li B, Fan R, Sun G, Sun T, Fan Y, Bai S, Guo S, Huang S, Liu J, Zhang H, et al. Flavonoids improve drought tolerance of maize seedlings by regulating the homeostasis of reactive oxygen species. Plant and Soil. 2021;461:389–405. doi: 10.1007/s11104-020-04814-8.

[70] Lugtenberg F, Kamilova. Plant-growth-promoting rhizobacteria. Annu Rev Microbiol. 2009;63(1):541–556. doi: 10.1146/annurev.micro.62.081307.162918. 19575558

[71] Munoz Aguilar JM, Ashby AM, Richards AJM, Loake GJ, Watson MD, Shaw CH. Chemotaxis of *Rhizobium leguminosarum* biovar phaseoli towards flavonoid inducers of the symbiotic nodulation genes. Microbiology. 1988;134(10):2741–2746. doi: 10.1099/00221287-134-10-2741.

[72] Zheng Y, Cao X, Zhou Y, Ma S, Wang Y, Li Z, Zhao D, Yang Y, Zhang H, Meng C, et al. Purines enrich root-associated pseudomonas and improve wild soybean growth under salt stress. Nat Commun. 2024;15(1):3520. doi: 10.1038/s41467-024-47773-9. 38664402 PMC11045775

[73] Sun X, Xu Z, Xie J, Hesselberg-Thomsen V, Tan T, Zheng D, Strube ML, Dragoš A, Shen Q, Zhang R, et al. Bacillus velezensis stimulates resident rhizosphere *Pseudomonas stutzeri* for plant health through metabolic interactions. ISME J. 2022;16(3):774–787. doi: 10.1038/s41396-021-01125-3. 34593997 PMC8483172

[74] Arif I, Batool M, Schenk PM. Plant microbiome engineering: expected benefits for improved crop growth and resilience. Trends Biotechnol. 2020;38(12):1385–1396. doi: 10.1016/j.tibtech.2020.04.015. 32451122

[75] Kim J-E, Woo O-G, Bae Y, Keum HL, Chung S, Woo, Sul J, Lee J-H. Enhanced drought and salt stress tolerance in *Arabidopsis* by *Flavobacterium crocinum* HYN0056T. J Plant Biol. 2020;63(1):63–71. doi: 10.1007/s12374-020-09236-8.

[76] Verbon EH, Liberman LM. Beneficial microbes affect endogenous mechanisms controlling root development. Trends Plant Sci. 2016;21(3):218–229. doi: 10.1016/j.tplants.2016.01.013. 26875056 PMC4772406

[77] Trivedi P, Leach JE, Tringe SG, Sa T, Singh BK. Plant-microbiome interactions: from community assembly to plant health. Nat Rev Microbiol. 2020;18(11):607–621. 32788714 10.1038/s41579-020-0412-1

[78] Wang N, Wang T, Chen Y, Wang M, Lu Q, Wang K, Dou Z, Chi Z, Qiu W, Dai J, et al. Microbiome convergence enables siderophore-secreting-rhizobacteria to improve iron nutrition and yield of peanut intercropped with maize. Nat Commun. 2024;15(1):839. doi: 10.1038/s41467-024-45207-0. 38287073 PMC10825131

[79] Patil JR, Mhatre KJ, Yadav K, Yadav LS, Srivastava S, Nikalje GC. Flavonoids in plant-environment interactions and stress responses. Discov Plants. 2024;1(1):68. doi: 10.1007/s44372-024-00063-6.

[80] Wen J, Wang Y, Lu X, Pan H, Jin D, Wen J, Jin C, Sahu SK, Su J, Luo X, et al. An integrated multi-omics approach reveals polymethoxylated flavonoid biosynthesis in citrus reticulata cv. Chachiensis. Nat Commun. 2024;15(1):3991. doi: 10.1038/s41467-024-48235-y. 38734724 PMC11088696

[81] Liu M, Li X, Liu Y, Cao B. Regulation of flavanone 3-hydroxylase gene involved in the flavonoid biosynthesis pathway in response to UV-B radiation and drought stress in the desert plant, *Reaumuria soongorica*. Plant Physiol Biochem. 2013;73(December):161–167. doi: 10.1016/j.plaphy.2013.09.016. 24121417

[82] Shomali A, Das S, Arif N, Sarraf M, Zahra N, Yadav V, Aliniaeifard S, Chauhan DK, Hasanuzzaman M. Diverse physiological roles of flavonoids in plant environmental stress responses and tolerance. Plants. 2022;11(22):3158. doi: 10.3390/plants11223158. 36432887 PMC9699315

[83] Ismail H, Dragišic Maksimovic J, Maksimovic V, Shabala L, Živanovic BD, Tian Y, Jacobsen S-E, Shabala S. Rutin, a flavonoid with antioxidant activity, improves plant salinity tolerance by regulating K+ retention and Na+ exclusion from leaf mesophyll in quinoa and broad beans. Funct Plant Biol. 2015;43(1):75–86. doi: 10.1071/FP15312. 32480443

