# Supplementary material for: A beneficial bacterium mitigates drought stress by upregulating the flavonoid biosynthetic pathway in Arabidopsis
Source: Plant Signal Behav. 2026 Mar 10;21(1):2639558. doi: 10.1080/15592324.2026.2639558 (PMC12987532; doi:10.1080/15592324.2026.2639558)

**Supplementary Table1:** **Primers used in RT-qPCR**

| **Gene name** | **AGI code** | **Forward** | **Reverse** |
| --- | --- | --- | --- |
| *CHS* | *AT5G13930* | TCAGGCGGAGTATCCTGACTA | CGTTTCCGAATTGTCGACTT |
| *FLS1* | *AT5G08640* | TCACATCGGCGATCAGATT | GGGAGGCTCCAAGAAAACC |
| *F3H* | *AT3G51240* | GCGTCGATATGGACCAAAAG | CTTGAGTCCGAGGGTGAGAT |
| *CHI* | *AT3G55120* | AGTTCCTCGCAATGTTCTGGA | TGCTCGTGTGGATCTGAGAA |
| *F3'H* | AT5G07990 | AGACATCGCTCAGCTTCCTT | TGGTGGATGAAGCCTGAAAT |
| *MYB11* | AT3G62610 | AGTCCAACGGCGAAGGAT | CAGCTCTTTCCACATCTCTTTAGC |
| *UBQ10* | *AT4G05320* | GAAGTTCAATGTTTCGTTTCATGT | GGATTATACAAGGCCCCAAAA |

**Supplementary Table 2:** Reactive Oxygen Species Related GO Biological Processes Enriched in PGPR+PEG versus Control Arabidopsis Plants

| **GO term** | **description** | **pval** | **padj** | **Genes** |
| --- | --- | --- | --- | --- |
| GO:0006979 | response to oxidative stress | 3.41E-15 | 7.66E-13 | FER1, HSP17.6C, ADH1, PRXIIB, P5CSA, HSP17.6B, LACS7 |
| GO:0000302 | response to reactive oxygen species | 6.85E-15 | 1.38E-12 | CAT2, FER1, HSP17.6C, ADH1, HSP17.6B, LACS7, CLPB1, GAPC1 |
| GO:0042542 | response to hydrogen peroxide | 4.48E-13 | 4.53E-11 | HSP17.6, EGY3, HSP17.4B, HSP70-8, GOLS1, HSP17.4A |
| GO:0034599 | cellular response to oxidative stress | 2.84E-06 | 5.53E-05 | PRXIIB, ATATH13, APXS, ATSRX, AAF, CSD1, APX3, MSRB1, GRXC5 |
| GO:0071450 | cellular response to oxygen radical | 0.00018 | 0.001924 | CSD1, FSD1, NTR2, MSD1, MYB75, FSD3, CCS |
| GO:0071451 | cellular response to superoxide | 0.00018 | 0.001924 | CSD1, FSD1, NTR2, MSD1, MYB75, FSD3, CCS |
| GO:0072593 | reactive oxygen species metabolic process | 0.000223 | 0.00233 | CAT2, ATMDAR1, PRXIIB, APXS, MDAR4, ALDH12A1, PAP26, AAF, PXG |
| GO:0000303 | response to superoxide | 0.00046 | 0.00418 | CSD1, FSD1, NTR2, MSD1, MYB75, FSD3, CCS |
| GO:0000305 | response to oxygen radical | 0.00046 | 0.00418 | CSD1, FSD1, NTR2, MSD1, MYB75, FSD3, CCS |


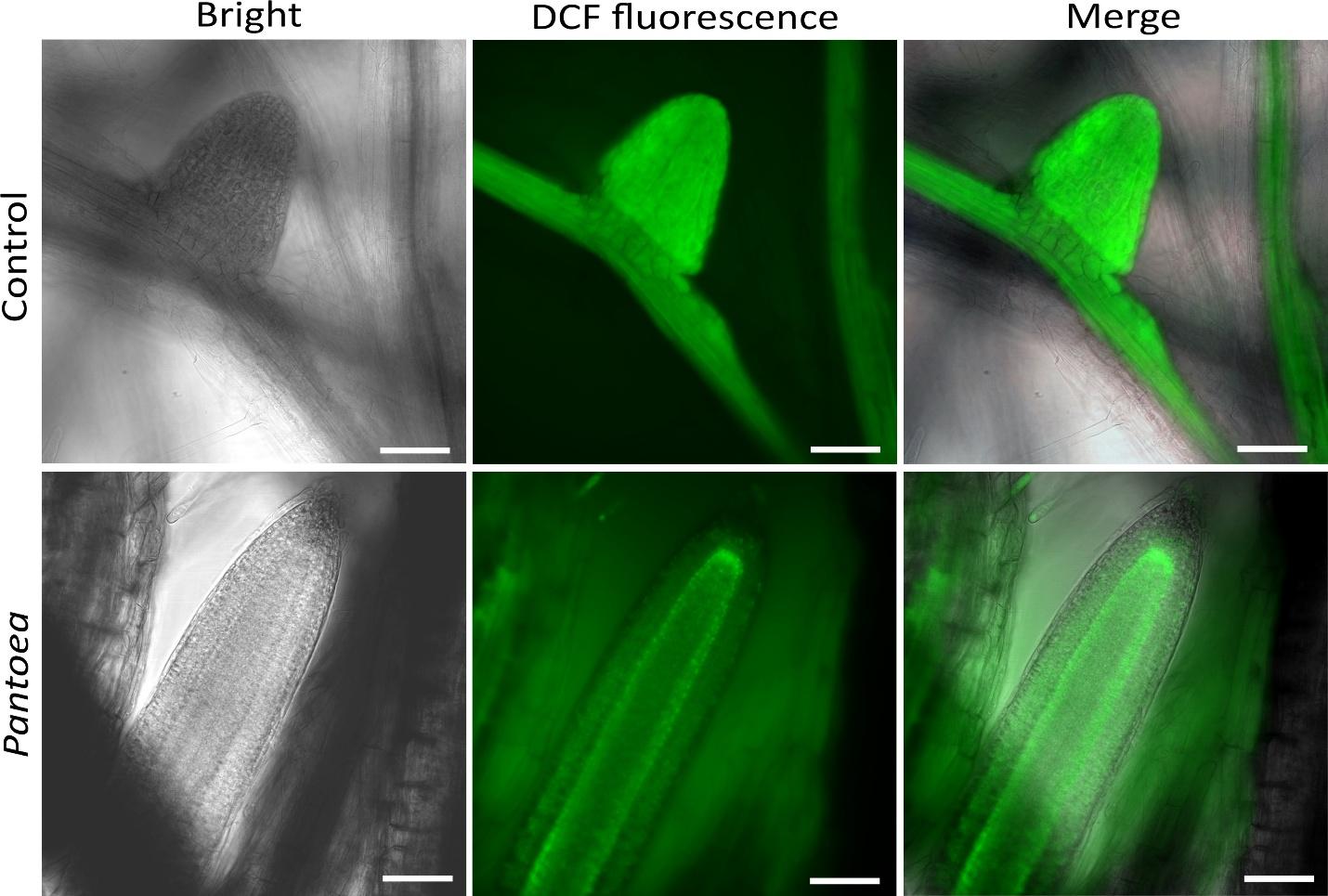


**Supplementary Figure 1. *Pantoea* sp. colonization modulates reactive oxygen species (ROS) accumulation in plant roots:** Representative confocal images of roots stained with H2DCFDA. Untreated control plant roots exhibit intense, highly localized DCF fluorescence (green) primarily at the site of emerging lateral roots. Plant roots inoculated with *Pantoea* species, displaying a more diffuse and altered distribution of ROS across the root tip. Scale bars indicate 50 µm.

**Supplementary Figure 2**: a) Diameter of rosettes in *Pantoea* inoculated and uninoculated plants under well-watered and drought stress conditions. ANOVA (Tukey’s multiple comparison test), *P* < 0.05.  b) Total chlorophyll content in *Col-0*, *fls-1*, *tt4-2*, and *omt1* in twenty-day old plants 10 days after drought stress. ANOVA (Tukey’s multiple comparison test). *P* < 0.05.  n=120. The experiment had 2 treatments and 4 plant genotypes. Each treatment had 3 replicates. FW= fresh weight. (PEG refers to the period when seedlings are transferred to MS containing 20% PEG 6000, Recovery refers to transfer from PEG media into PEG-free MS + 1.5% sucrose (recovery media). [Drought refers to the period when seedlings are transferred to MS containing 20% PEG 6000, Recovery refers to transfer from PEG media into PEG-free MS + 1.5% sucrose (recovery media)].


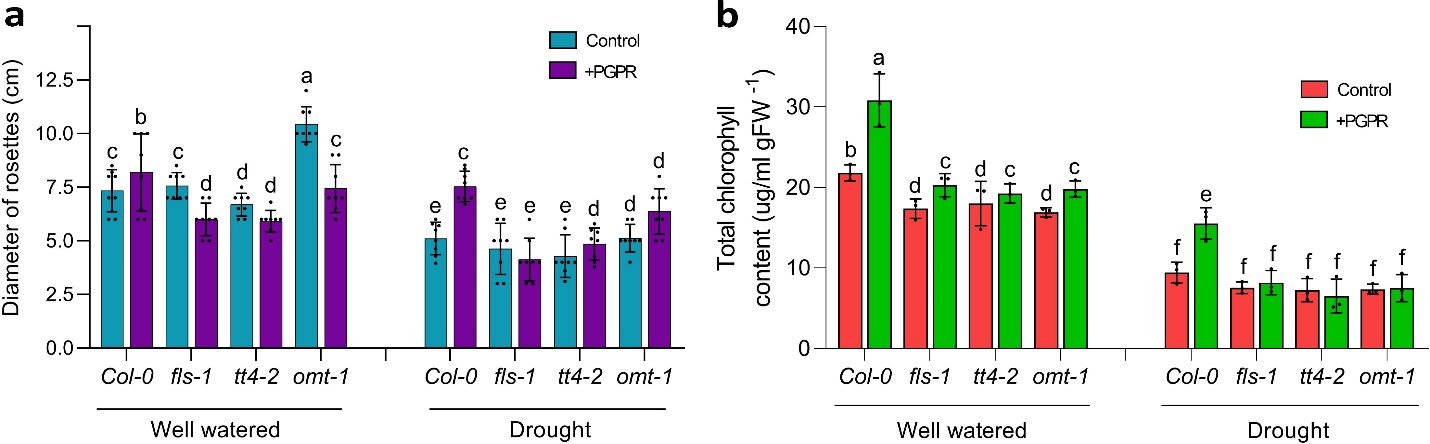


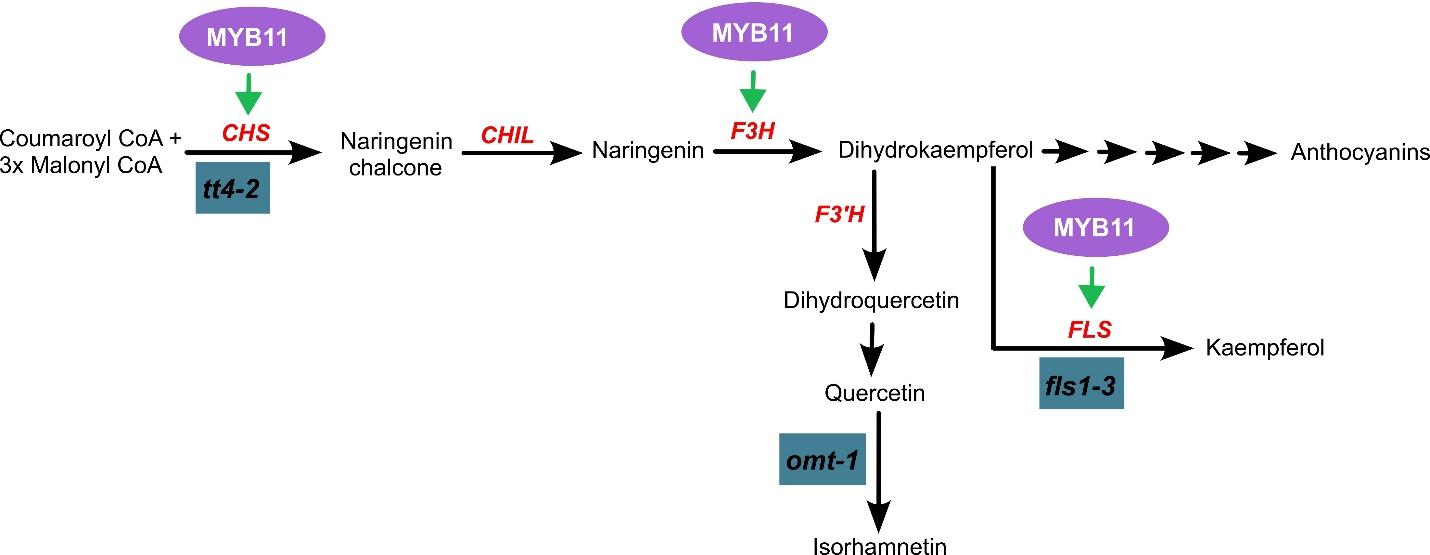


**Supplementary Figure 3:** A simplified flavonoid biosynthesis pathway showing location of different genes (in red) quantified in this study. The green arrows point to genes that are directly acted upon by the MYB11 TF. Mutants used in this work are indicated by blue boxes, *tt4-2*, *fls1-3* and *omt1*. TF: transcription factor.

**Supplementary Figure 4: Arabidopsis root colonization by *Pantoea* sp under PEG-induced stress in vitro** (a) Quantification of bacterial colonization in *Col-0* and flavonoid pathway mutants (*fls-1, tt4-2, omt-1*) expressed as CFU per g fresh weight following PGPR inoculation. Mock-inoculated controls are shown for each genotype. Bars represent mean ± SEM (n = 50). ****P < 0.0001 by one-way ANOVA. (b) *Pantoea* sp colonization (CFU/g fresh weight) of Col-0 seedlings in ½ MS (0 days), transferred to PEG containing MS media for drought stress (3 days and 5 days), and then placed in ½ MS (recovery for 5 days). Control seedlings were mock-inoculated. Points show mean ± SEM. Asterisk indicates a significant difference relative to 0 days’ time point (*P < 0.05) by one-way ANOVA.


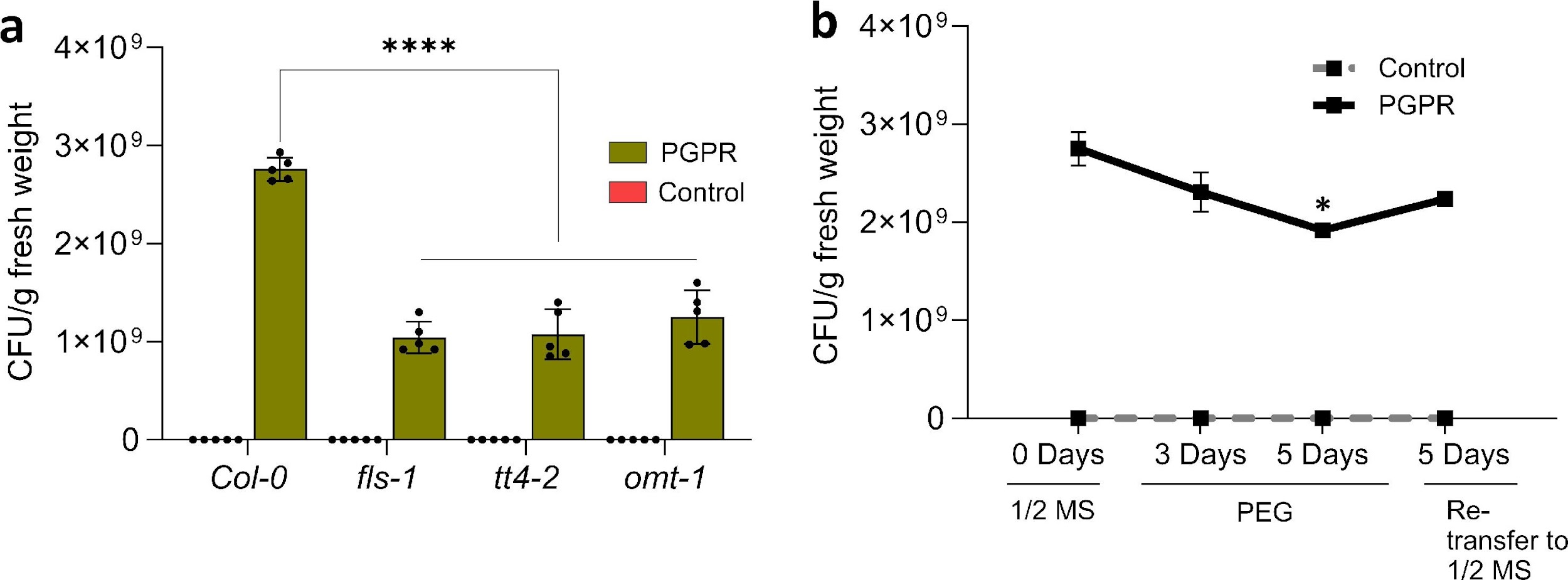

Supplement: Supplementary material [file KPSB_A_2639558_SM1495.docx]
